# Quantitative patterns of motor cortex proteinopathy across ALS genotypes

**DOI:** 10.1186/s40478-020-00961-2

**Published:** 2020-07-02

**Authors:** Matthew Nolan, Connor Scott, Menuka Pallebage Gamarallage, Daniel Lunn, Kilda Carpenter, Elizabeth McDonough, Dan Meyer, Sireesha Kaanumalle, Alberto Santamaria-Pang, Martin R. Turner, Kevin Talbot, Olaf Ansorge

**Affiliations:** 1 Nuffield Department of Clinical Neurosciences, University of Oxford, Level 1, West Wing, John Radcliffe Hospital, Oxford, OX3 9DU UK; 2 Department of Statistics, University of Oxford, Oxford, UK; 3 GE Research, Niskayuna, NY USA

**Keywords:** ALS, FTD, Selective vulnerability, TDP-43, *C9ORF72*

## Abstract

Degeneration of the primary motor cortex is a defining feature of amyotrophic lateral sclerosis (ALS), which is associated with the accumulation of microscopic protein aggregates in neurons and glia. However, little is known about the quantitative burden and pattern of motor cortex proteinopathies across ALS genotypes. We combined quantitative digital image analysis with multi-level generalized linear modelling in an independent cohort of 82 ALS cases to explore the relationship between genotype, total proteinopathy load and cellular vulnerability to aggregate formation. Primary motor cortex phosphorylated (p)TDP-43 burden and microglial activation were more severe in sporadic ALS-TDP disease than C9-ALS. Oligodendroglial pTDP-43 pathology was a defining feature of ALS-TDP in sporadic ALS, C9-ALS and ALS with *OPTN*, *HNRNPA1* or *TARDBP* mutations. ALS-FUS and ALS-SOD1 showed less cortical proteinopathy in relation to spinal cord pathology than ALS-TDP, where pathology was more evenly spread across the motor cortex-spinal cord axis. Neuronal pTDP-43 aggregates were rare in GAD67+ and Parvalbumin+ inhibitory interneurons, consistent with predominant accumulation in excitatory neurons. Finally, we show that cortical microglia, but not astrocytes, contain pTDP-43. Our findings suggest divergent quantitative, genotype-specific vulnerability of the ALS primary motor cortex to proteinopathies, which may have implications for our understanding of disease pathogenesis and the development of genotype-specific therapies.

## Introduction

The term ‘selective vulnerability’ describes the differential susceptibility of cells or anatomically defined systems to disease pathomechanisms. In the context of neurodegenerative disease, this can be further delineated as vulnerability to cellular proteinopathy or vulnerability to degeneration itself; a relationship which cannot be assumed to be linear, as microscopically visible aggregate formation may be an indicator of a successful cellular response to proteotoxicity, dependent on cell-specific concentration-dependent thresholds for protein precipitation [3, 16, 28, 73]. In amyotrophic lateral sclerosis (ALS), this selectivity is particularly apparent as functionally distinct motor neuron subtypes in close anatomical proximity are variably affected [13, 56], however whether this reflects cell-intrinsic vulnerabilities or connectivity, or both, remains unclear. Around 10% of ALS cases are caused by autosomal dominant mutations of varying penetrance to one or more known genes including *C9ORF72* (C9-ALS) [17, 64], *SOD1* (ALS-SOD1) [68], *FUS* (ALS-FUS) [43] and *TARDBP* [81], with some mutations appearing to predispose patients towards an upper (UMN) or lower (LMN) motor neuron predominant phenotype [29, 60, 65, 79]. The primary neuropathological observation in ~ 95% ALS cases is the cytoplasmic mislocalization and aggregation of hyper-phosphorylated TDP-43 (pTDP-43) within neurons and glia (ALS-TDP) [54].

While ALS patients commonly present with a combination of LMN and pyramidal signs, the nature of disease initiation and progression between the spinal cord and cortex remains unclear. In the ‘dying forward’ hypothesis, motor cortex dysfunction associated with anterograde glutamate-mediated excitotoxicity precedes LMN dysfunction [19, 24]. However, attempts to identify a histological correlate for this hypothesis using human tissue have produced conflicting results [30, 48, 55], compounded by neuropathological assessment often being confined to subjective readouts of disease burden which are a source of bias and potentially increase type I error. Digital image analysis algorithms have previously been used to accurately quantify TDP-43 pathology more objectively [35, 86], and the combination of quantitative disease indicators and genetic architecture can be used to produce neuropathological endophenotypes independent of relatively crude clinical readouts, which may not be up-to-date during the terminal phase of the illness [2, 18, 53].

Surprisingly, there have been no systematic studies attempting to define and quantify the total proteinopathy burden and its cellular pattern in the primary motor cortex across ALS genotypes since the discovery of the main ALS driver genes and their protein products. We hypothesized that the ratio of motor cortex to spinal cord proteinopathy burden is not uniform across genotypes and that not all cell types are equally affected by protein aggregates. For example, we therefore sought to clarify if inhibitory interneurons, excitatory pyramidal neurons, or oligo−/astroglia preferentially accumulate protein aggregates in the motor cortex. Here, we use quantitative digital image analysis in conjunction with multilevel statistical modelling to show that primary motor cortex phosphorylated (p)TDP-43 burden and microglial activation was more severe in sporadic ALS-TDP disease than C9-ALS, that oligodendroglial pTDP-43 pathology was a defining feature of all genetic subgroups of ALS-TDP, that ALS-FUS and ALS-SOD1 show less cortical proteinopathy in relation to spinal cord pathology than ALS-TDP, and that neuronal pTDP-43 aggregates are rare in GAD67+ inhibitory interneurons consistent with predominant accumulation in excitatory neurons. Our data from an independent ALS cohort thus contribute to our understanding of motor cortex neuropathology across diverse ALS genotypes.

## Materials and methods

### Cases

Cases were included if the primary clinical presentation was ALS as judged by an experienced neurologist with a special interest in ALS. Presentation with frontotemporal dementia (FTD) was an exclusion criterion. A further inclusion criterion was availability of definitive primary motor cortex (defined by the presence of Betz cells) and lumbar spinal cord blocks. Post-mortem human brain tissue in the form of 10 μm sections was obtained from the Oxford Brain Bank, the MRC London Neurodegenerative Diseases Brain Bank and the Sheffield Brain Tissue Bank. Consent and ethical approval for the use of tissue was provided by the generic REC approval of each research tissue bank (Oxford - 15/SC/0639, MRC London - 08/MRE09/38, Sheffield - 08/MRE00/13). All cases were diagnosed post-mortem by an experienced neuropathologist and confirmed genetically via Sanger or whole-exome sequencing. For the purposes of this study, we therefore use the term ‘sporadic’ to refer to cases with no high-penetrance mutations in known ALS genes and an absence of characteristic genetically-linked pathology. Whole-exome sequencing failed to find a causative mutation in one patient (case 8), but was neuropathologically confirmed as ALS-FUS through the presence of characteristic FUS pathology. Case 6 also exhibited a Y374X *TARDBP* variant which is predicted to be damaging, but displayed no TDP-43 pathology [40] and overall the case more closely resembles the juvenile-onset ALS associated with ALS-FUS. Cases caused by *CHMP2B*, *HNRNPA1*, *OPTN* and *TARDBP* mutations are present in < 1% ALS patients, and we were therefore unable to obtain a significant number of these genotypes, however they were included because of their potential for interesting comparisons and clarification of their pathotype. Demographic and clinical details (where available) of all cases are included in the supplementary material and summarised in Table 1.

**Table 1:** Summary of all cases used in this study. See supplementary data for subgroup analyses

Genotype	***n*** =	With FTD	Sex		Age (Years)			PMD (Hours)			Fixation (Weeks)		
			M	F	Range	Mean	Std. Dev.	Range	Mean	Std. Dev.	Range	Mean	Std. Dev.
**Control**	17	0	10	7	36–95	62.5	14.0	18–73	42.5	16.0	0.2–32	7.1	9.2
**Sporadic**	39	7	21	18	44–90	65.5	11.7	24–120	47.7	22.5	0.2–28	5.9	8.5
***C9ORF72***^a^	18	5	11	7	42–76	60.6	9.4	4–96	47.5	30.5	0.2–17	7.5	4.8
***SOD1***^b^	11	0	3	8	34–78	55.9	14.7	5–96	39.0	31.9	3–20	11.8	5.8
***FUS***^c^	9	0	4	5	18–64	37.9	15.2	19–72	47.0	19.5	2–45	12.6	13.7
***CHMP2B***^d^	2	0	1	1	51–69	60.0	12.7	12–22	17.0	7.1	3–4.5	3.8	1.1
***HNRNPA1***^e^	1	0	0	1	62	62.0	–	48	48.0	–	12.0	12.0	–
***OPTN***^f^	1	0	1	0	54	54.0	–	72	72.0	–	12.0	12.0	–
***TARDBP***^g^	1	0	1	0	57	57.0	–	48	48.0	–	9.0	9.0	–

^a^*C9ORF72* – Chromosome 9 open reading frame 72; ^b^*SOD1* – Superoxide dismutase 1; ^c^*FUS* – Fused-in-sarcoma; ^d^*CHMP2B* – Charged multivesicular body protein 2B; ^e^*HNRNPA1* – Heterogeneous nuclear ribonucleoprotein A1; ^f^*OPTN* – Optineurin; ^g^*TARDBP* – TAR DNA-binding protein. PMD = post-mortem delay

### Tissue sampling

10 μm sections containing underlying white matter were cut from archival formalin-fixed paraffin-embedded (FFPE) blocks from the primary motor cortex, corresponding to the ‘hand knob’ region of the homunculus where available. 5 μm sections from the same region were used in multiplexed-immunofluorescence experiments. 10 μm sections of FFPE lumbar spinal cord were also cut from each case.

### DAB-immunohistochemistry

De-identified sections from sporadic (*n* = 19), *C9ORF72* (*n* = 16), *SOD1* (*n* = 11), *FUS* (*n* = 9), *CHMP2B* (*n* = 2), *TARDBP* (*n* = 1), *HNRNPA1* (*n* = 1), *OPTN* (*n* = 1) and control (*n* = 11) cases (‘s-IHC cohort’) were immunohistochemically stained according to standard protocols. Primary antibody clones (Table 2) were chosen based on specificity demonstrated in protein expression databases (e.g Human Protein Atlas) as well as validation in previous studies. Targets were visualised using HRP-conjugated secondary antibodies (Dako Envision+ kit, Agilent, USA), which were incubated for 1 h at room temperature before detection using DAB substrate for 10 min. Extensive quality-control experiments were conducted with each antibody prior to batch staining. Specifically, we tested fixation-dependency of the reaction products and qualitatively screened for non-specific staining. The pTDP-43 antibody performed equally well in short- and long-fixed material, with no background staining. In contrast, FUS antibody showed only weak signal in long fixation cases and SOD1 antibody (SEDI) showed very strong non-specific astroglial staining in control tissue and missed some protein aggregates in SOD1 cortex that were clearly visible on HE sections (e.g. hyaline conglomerate inclusions). These antibodies therefore served to support the diagnosis of ALS-FUS and ALS-SOD1 cases respectively, but were not suitable for quantitative digital analysis. We did not have access to anti-dipeptide antibodies that performed robustly across the cohort. However, p62 proved an excellent generic stain for ALS protein aggregates (including dipeptides) and was used as a read-out for pan-aggregation load in the selected ROIs [1, 23, 42]. None of the primary motor cortices contained tau, alpha-synuclein or amyloid-beta aggregates. For chromogenic double IHC, pTDP-43 and either GAD67, Olig2 or NeuN primary antibodies (Table 2) were applied to a subsection of the cohort (control *n* = 3, ALS *n* = 7; Supplementary material). These were sequentially stained using pTDP-43 primary antibody with HRP-DAB detection, followed by either GAD67, Olig2 or NeuN primary antibody and goat anti-rabbit secondary antibody conjugated to alkaline phosphatase (1:1000, Dako, cat# D0487) with FastRed substrate detection. To assess the relative extent of upper versus lower motor neuron involvement, an additional three sections from the lumbar spinal cord region were cut at 10 μm from each case and stained with pTDP-43, CD68 and SMI-312. Positive and negative controls were included for each antibody. No staining was seen when the primary antibody was omitted. Counterstaining was performed using Coles haematoxylin. Giant pyramidal cells of Betz define the human primary motor cortex. For all cases therefore, an additional section was stained using haematoxylin and eosin (H&E) to confirm the presence of Betz cells in layer Vb. Stained sections were then dehydrated, cleared and mounted (Histomount, Thermo-Fisher Scientific, Germany) and viewed using a Zeiss Primo Star microscope (Zeiss, Oberkochen, Germany).

**Table 2:** Primary antibody clones used in this study

Antigen	Supplier	Catalogue #	Host	Clonality	Dilution	
					IHC	MxIF
pTDP-43^a^	Cosmo-bio	TIP-PTD-M01	Mouse	Monoclonal	1:40000	1:10000
p62^b^	Abcam	ab91526	Rabbit	Polyclonal	1:1000	–
FUS^c^	Sigma-Aldrich	HPA008784	Rabbit	Polyclonal	1:300	–
SOD1^d^	StressMarq Bio	SPC-206	Rabbit	Polyclonal	1:1000	–
HuC/D^e^	Santa Cruz	sc-28,299	Mouse	Monoclonal	–	1:400
SMI312^f^	Sternberger monoclonals	837,904	Mouse	Monoclonal	1:2000	–
NeuN	Abcam	ab177487	Rabbit	Monoclonal	1:2000	–
Parvalbumin	Swant	235	Mouse	Monoclonal	1:7500	1:5000
GFAP^g^	Sigma-Aldrich	C9205	Mouse	Monoclonal	–	1:800
Olig2^h^	Abcam	ab109186	Rabbit	Monoclonal	1:400	–
TPPP/p25^i^	Win-Fest Ltd	–	Mouse	Monoclonal	1:2000	–
Iba1^j^	WAKO	019–19,741	Rabbit	Polyclonal	–	1:400
CD68^k^	Dako	M0876	Mouse	Monoclonal	1:200	–
	ThermoFisher	MS397	Mouse	Monoclonal	–	1:800
Collagen IV	Merck Millipore	MAB3326	Mouse	Monoclonal	–	1:4000
OPTN^l^	Proteintech	10,837–1-AP	Rabbit	Polyclonal	1:3000	–
GAD67^m^	Merck Millipore	MAB5406	Rabbit	Monoclonal	1:1000	–

^a^pTDP-43 - Phosphorylated transactive DNA response protein 43 kDa; ^b^p62 – Sequestosome; ^c^FUS – Fused-in-sarcoma; ^d^SOD1 – Superoxide dismutase 1; ^e^HuC/D – ELAV-like protein 4; ^f^SMI312 – Panaxonal Neurofilament marker; ^g^GFAP – Glial fibrillary acidic protein; ^h^Olig2 – Oligodendrocyte transcription factor 2; ^i^TPPP – Tubulin polymerization-promoting protein; ^j^Iba1 – Ionized calcium-binding adapter molecule 1; ^k^CD68 – Cluster of differentiation 68; ^l^OPTN – Optineurin; ^m^GAD67 – Glutamate decarboxylase 67

### Quantification of single-immunohistochemistry

**Fig. 1:**
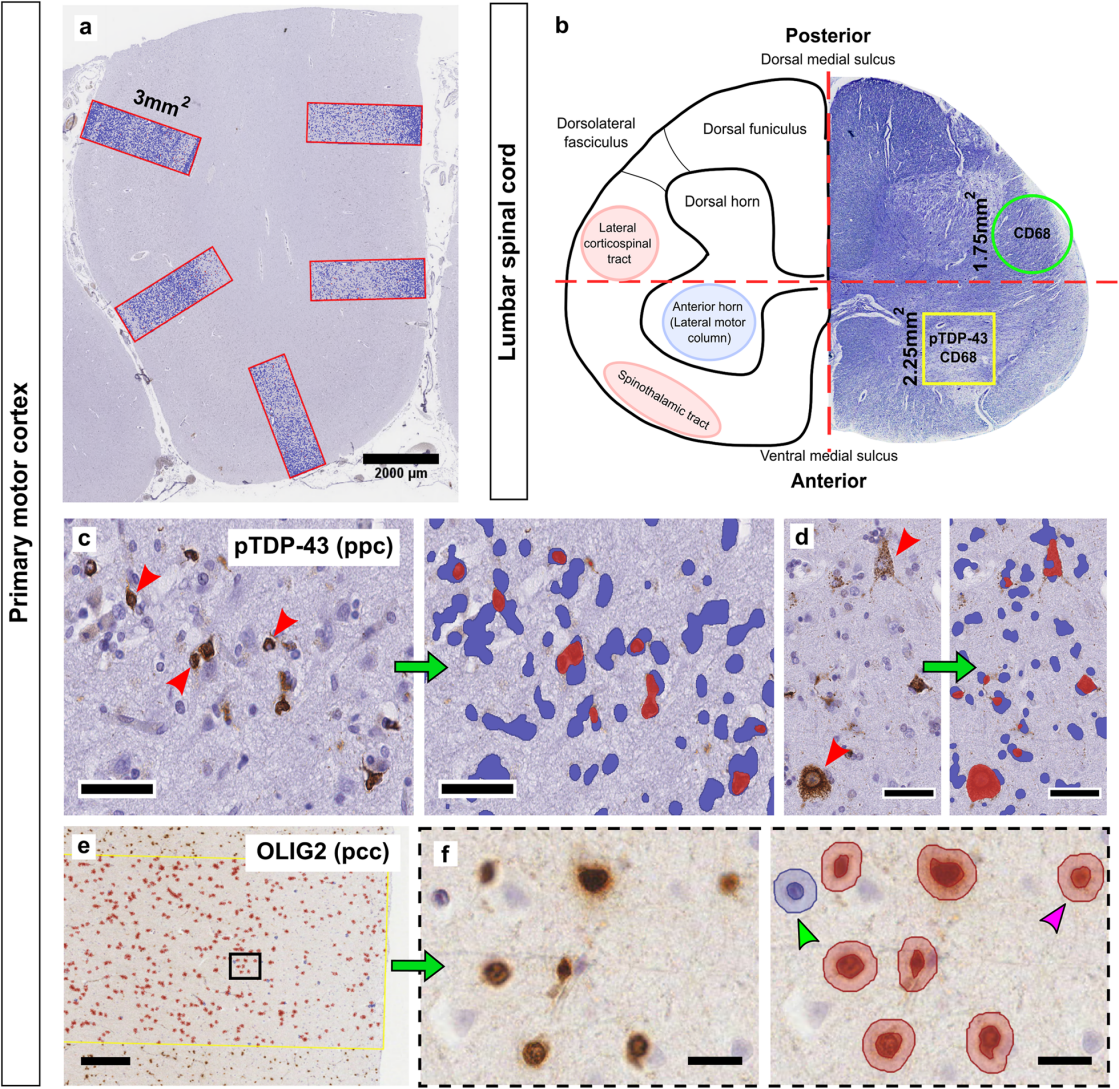
Quantitative assessment of pathology in the ALS primary motor cortex and spinal cord using optimised digital image analysis. IHC staining was quantified in the motor cortex using DAB optical-density based algorithms on five ROI, each measuring 3mm^2^. ROI were spread evenly around a single gyrus where the top edge of the ROI was at the pial surface (**a**). CD68 and pTDP-43 pathology was also quantified in the anterior horn and corticospinal tract of the lumbar spinal cord (**b**, yellow and green box respectively), using guidelines bisecting the section in each direction across the central canal. An optimised positive pixel count (ppc) algorithm is able to distinguish variable pTDP-43 morphologies including glial (**c**, red arrows) and neuronal (**d**, red arrows). A positive cell count (pcc) algorithm counts the number of Olig2+ oligodendrocytes (**e**, black box = area of f), and defines positive cells by DAB-threshold with high specificity (**f**, pink arrow highlights DAB-positive cell, green arrow highlights DAB-negative cell). Scale bars where not indicated (μm): c,d = 50; e = 250; f = 20

Slides were digitally scanned using the Aperio ScanScope AT Turbo system (Leica Biosystems, Germany) and analysed using QuPath software [4]. For pathological quantification of the primary motor cortex, regions of interest (ROI) measuring 1000 μm × 3000 μm were defined and quantified using thresholding and algorithms optimized for each stain. Optimisation of algorithms included adjustment for nuclear/cytoplasmic area, fragmentation of nuclei, pixel size and DAB thresholding. The algorithms themselves are included in the supplementary material. CD68, TPPP/p25 and Olig2 were quantified using a positive cell count (pcc; Fig. 1e-f). Because of the morphological heterogeneity of pTDP-43 and p62 pathology, these were quantified using a positive pixel count (ppc) tool (Fig. 1c,d). Positive pixel count approaches have been used and validated in other human post-mortem studies [35, 86]. Both methods measured the number of positive cells/pixels above a defined DAB optical density (OD) threshold, which was set by plotting mean DAB OD against count for all detections in a given ROI and selecting the lowest OD required to produce minimal false positive/negatives. After optimisation, algorithms were applied consistently throughout. Five ROI were assessed per slide, and placed where the short edge of each ROI was touching the pial surface whilst parallel with underlying white matter. ROI were placed evenly around a single gyrus on each slide, and measurements averaged across the five ROI (Fig. 1a). For CD68, three additional ROI within the subcortical white matter of a single gyrus were also defined and measurements averaged. For lumbar spinal cord sections, one circular ROI measuring 1.75mm^2^ and one square ROI measuring 2.25mm^2^ was placed in the lateral corticospinal tract region and anterior horn region respectively, on each side of a single lumbar spinal cord slice (Fig. 1b) and measurements averaged. CD68 and pTDP-43 were quantified in the spinal cord using the same algorithms as the motor cortex. The area of 10 anterior horn neurons (5 from each half of the spinal column) from the lumbar region of each case were measured by drawing around the perimeter of each neuron at × 40 mag. Measurements were then averaged and log transformed for statistical testing.

For oligodendrocyte analysis, criteria for designation as an oligodendrocyte were presence of characteristic round nucleus, chromatin structure and nucleolus, thin rim or perinuclear clear cytoplasm and position as grey matter satellite or subcortical white matter oligodendrocyte. Oligodendroglial TDP-43 inclusions have a characteristic ‘comma’ or ‘wisp-like’ appearance. Neuronal criteria were size (> 10 μm) and shape (triangular or oval), position in cortical layer, presence of peripheral Nissl substance, and a round nucleus with pale homogenous nucleoplasm and a single dark nucleolus.

All analyses were conducted blind to case details including genotype and other IHC results.

### Multiplexed immunofluorescence

Multiplex experiments were conducted on a subset of cases, all with a short (< 48 h) fixation protocol, as most antibodies suitable for multiplex experiments did not work after long (> 4 weeks) fixation. Multiplexed immunofluorescence (MxIF) staining and imaging was performed at GE Research using previously described methodologies [25], optimized for short-fixed FFPE neurological specimens. In brief, antibodies (Table 2) were applied two or three at a time across nine sequential staining-and-dye-deactivation rounds to a subsection of our cohort (sporadic *n* = 18, *C9ORF72 n* = 3, controls *n* = 5; Supplementary material). Antigens were detected either by directly fluorophore-conjugated antibodies, by pre-bound fluorophore-labelled antibody binders, or using primary antibody followed by fluorophore-labelled secondary antibodies. The staining of each antibody was imaged on separate channels after each staining round, and DAPI imaging of nuclei was collected in all rounds. Images were collected on custom Olympus IX81 inverted microscopes (Olympus, Tokyo, Japan) using 20x objectives, with multi-round image acquisition driven by automated, custom software. Images from different staining rounds were co-registered using DAPI staining (US Patent no. US8369600B2, 2013) [15] and then autofluorescence signal was removed by subtracting an earlier, unstained image from each corresponding stained image [84]. ROIs for MxIF acquisition were selected manually on the whole-tissue tissue sections to cover from the pial surface to the subcortical white matter of each case. Images were reviewed as a single panel composed of ~ 15 stitched fields of view per case at the University of Oxford using a custom plugin for ImageJ [71] enabling review of the multi-channel MxIF image data.

### Statistical analysis

Statistical analysis and graphing was performed using a combination of GraphPad Prism (California, USA) and multi-level generalized linear modelling (GLM) [52, 83] in RStudio (Boston, USA). The procedure used is analogous to a 3-way ANOVA where each individual *Case* is a block, *Protein* is a grouping variable and *Genotype* is a treatment, but in order to avoid over-parameterisation *Case* was incorported as a random effect in a 2-level multilevel model; thus within-*Case* correlation is accounted for. Goodness-of-fit chi-squared test revealed the data was negatively-binomially distributed. If the mean *Count* is *μ*, a negative-binomial GLM fits the model:

$$ \log \mu =\alpha +\sum \limits_i{\beta}_i{Genotype}_i+\sum \limits_j{\beta}_j{Protein}_j+\sum \limits_{i,j}{\beta}_{ij}{Genotype}_i:{Protein}_j $$

Unpaired Welch’s *t*-test was used to test oligodendrocyte pathologies. D’Agostino and Pearson method was used to assess normality prior to testing correlation coefficients. Scatterplots are formatted on a logarithmic base 2 scale and labelled as exponents of the base value. Levels for quoted confidence intervals are set at 95% and *p*-values in the figures are indicated as follows: * *p* < 0.05, ** *p* < 0.01, *** *p* < 0.001, **** *p* < 0.0001.

### Data availability

The dataset(s) supporting the conclusions of this article are included within the article and its additional files.

## Results

### Pathology of the ALS primary motor cortex is highly variable both within and across the spectrum of ALS genotypes

**Fig. 2:**
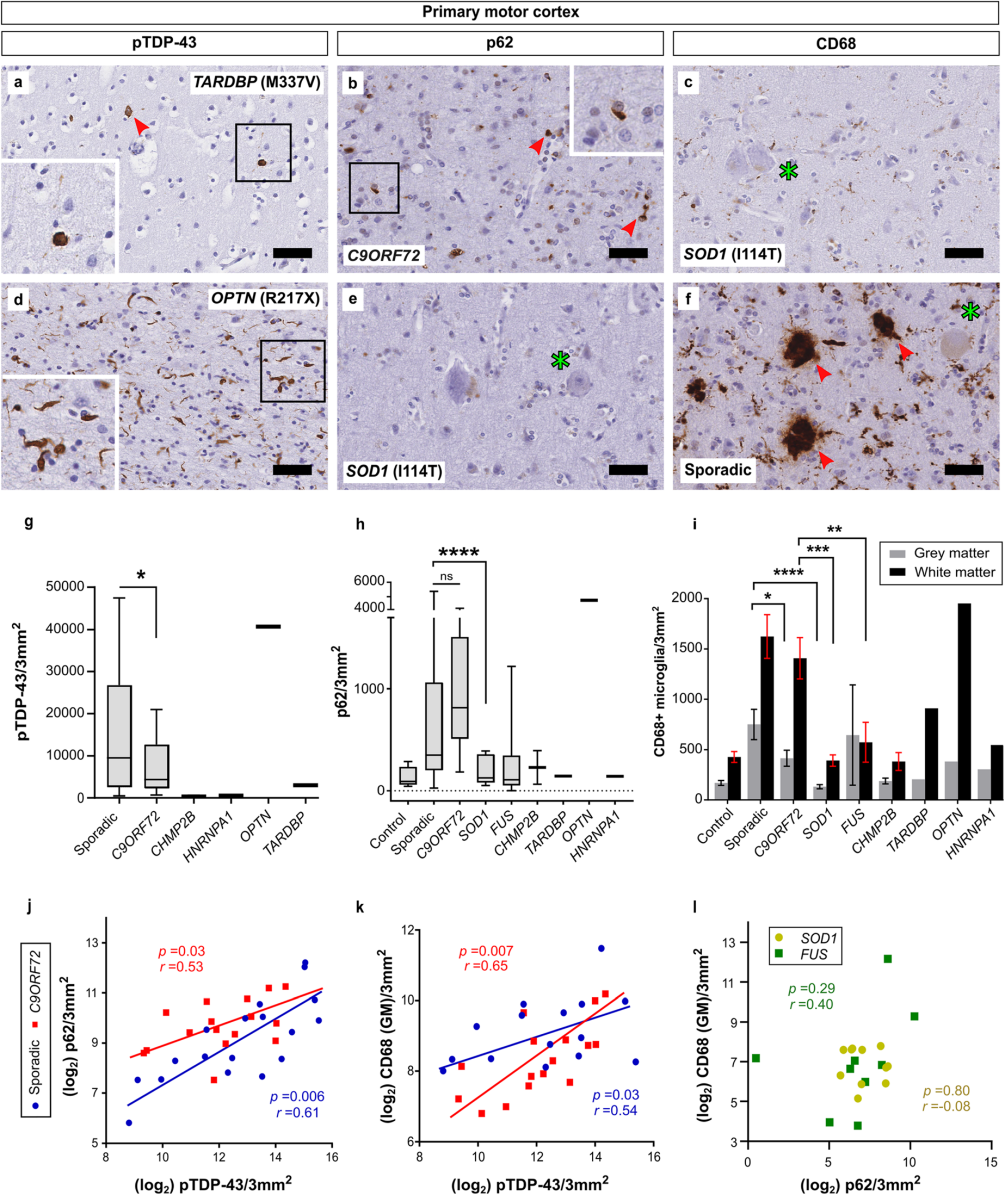
Pathology of the ALS primary motor cortex is variable both within and across the genotypic spectrum of disease. Relatively little pTDP-43 aggregation was found in a single *TARDBP* mutation case (**a**), but this was severe in an *OPTN* mutation case (d; see also supp. Figure 2). Insets highlight variance of pTDP-43 morphology between genotypes. Average highest pTDP-43 deposition was seen in sporadic cases, which was statistically higher than in C9-ALS (**g**). Quantification of p62 (**b**, **e**, **h**). Levels of p62 correlated with pTDP-43 in sporadic cases but less so in *C9ORF72* disease, reflective of the existence of p62-positive dipeptide repeat protein species unique to C9-ALS (**h** and **j**). Cortical microglial activation was highly variable between genotypes (**i**), and in some cases there was evidence of severe nodular neuronophagia surrounding layer V neurons (**f**). Grey matter CD68 correlated with the extent of pTDP-43 deposition (**k**) in both sporadic and *C9ORF72* cases, but this relationship was not recapitulated using p62 and CD68 in *SOD1*/*FUS* cases (**l**). Arrows highlight pathology, asterisks highlight Betz cells. *r* correlations = Pearson (**j**) or Spearman (**k**, **l**), results as on figure. Bars in (**i**) represent means and SEM. Best-fit lines are manually added for illustrative purposes. All scale bars = 50 μm

To assess the relative extent of pathology within the primary motor cortex across ALS genotypes, we performed immunohistochemical staining on our cohort (‘s-IHC cohort’; see supplementary material) using antibodies for the common pathological markers pTDP-43, p62, and CD68 (Fig. 2; Table 3).

**Table 3:** Primary motor cortex and spinal cord single-IHC results

			Primary motor cortex				Spinal cord			
							Anterior horn			Corticospinal tract
Genotype	n =	Statistic	Positive pixel count/3mm^2^		Positive cell count/3mm^2^		Neuron size (μm^2^)	PPC/2.25mm^2^	PCC/2.25mm^2^	Positive cell count/1.75mm^2^
			pTDP-43	p62	CD68 (GM^b^)	CD68 (WM^c^)		pTDP-43	CD68	CD68
**Control**	11	Mean	–	139.0	164.3	399.4	2163.0	–	271.2	140.3
		95% CI^a^	–	76.0–254.0	93.2–289.6	226.6–703.8	1429.0–2897.0	–	174.3–421.9	90.1–218.3
**Sporadic**	19	Mean	14,662.3	989.3	707.7	1613.6	763.9	11,626.9	1104.5	993.6
		95% CI	9088.2–23,655.1	653.4–1497.9	477.6–1048.5	1089.1–2390.7	495.5–1032.0	7612.5–17,758.1	850.5–1434.3	765.1–1290.4
***C9ORF72***	16	Mean	7143.1	1020.8	389.3	1353.8	794.9	10,068.0	1228.3	1179.0
		95% CI	4300.9–11,863.4	649.6–1604.1	253.8–597.2	882.6–2076.5	494.4–1095.0	6346.1–15,972.9	917.1–1645.0	880.3–1579.0
***SOD1***	11	Mean	–	186.1	123.6	366.0	469.5	–	987.3	837.7
		95% CI	–	107.8–321.0	73.9–206.8	219.0–611.9	258.1–681.0	–	694.1–1404.4	588.9–1191.6
***FUS***	9	Mean	–	264.1	471.0	490.0	404.4	–	1489.1	782.0
		95% CI	–	144.5–482.7	267.3–829.9	278.1–863.4	190.9–618.0	–	1008.7–2198.3	529.7–1154.5
***CHMP2B***	2	Mean	259.5	229.0	188.5	382.5	217.5	5673.0	1251.0	679.0
		95% CI	–	–	–	–	71.38–363.6	–	–	–
***TARDBP***	1	Mean	2970.0	144.0	205.0	910.0	853.0	7779.0	881.5	649.0
		95% CI	–	–	–	–	–	–	–	–
***HNRNPA1***	1	Mean	463.0	142.0	305.0	547.0	325.0	10,975.0	2149.0	1189.0
		95% CI	–	–	–	–	–	–	–	–
***OPTN***	1	Mean	40,668.0	3514.0	383.0	1954.0	1363.0	1649.0	28.5	85.0
		95% CI	–	–	–	–	–	–	–	–

^a^Confidence interval; ^b^Grey matter; ^c^White matter; ppc = positive pixel count; pcc = positive cell count

Morphology and severity of pTDP-43 was highly variable, even within cases of the same genotype. No pTDP-43 staining was detected in any of the *SOD1*, *FUS* or control cases. A formal goodness-of-fit test of a negative-binomial GLM fit for pTDP-43 staining was satisfactory (χ^2^(32) = 39.577, *p* = 0.167). On average, pTDP-43 aggregate burden was moderately but significantly higher in sporadic cases than *C9ORF72* disease (*p* = 0.043) (Fig. 2g). Notably, pTDP-43 pathology was relatively sparse across all layers in the single heterozygous *TARDBP* (M337V) mutation case analysed, but was widespread and severe in homozygous ALS-*OPTN* (R217X), particularly within layer II and V and at the grey/white matter border (Fig. 2a,d; Supp Figure 2).

Microglia are the innate immune cells of the central nervous system and robustly express the transmembrane glycoprotein CD68 in response to inflammatory injury as part of an ‘activated’ phenotype, a process which is commonly implicated in the pathogenesis of ALS (for review, see: [49, 62]). CD68 staining was therefore performed to assess the relative levels of microglial activation in both the grey and white matter within the primary motor cortex across our cohort (Fig. 2c, f, i). The relative extent of CD68 staining between the subcortical white matter and grey matter correlated in all genotypes (Supp. Figure 3). However, microglial activation was higher within the subcortical white matter than cortical grey matter in nearly all genotypes analysed (Fig. 2i). Differences in grey matter CD68 staining were significant between genotypes; staining was higher in sporadic cases than *C9ORF72* (*p* = 0.037) and *SOD1* cases (*p* = < 0.0001). In the subcortical white matter, CD68 expression was significantly higher in sporadic ALS-TDP cases than *SOD1* (*p* = < 0.0001) and *FUS* cases (*p =* 0.0006), and also significantly higher in *C9ORF72* cases than *SOD1* cases (*p* = 0.0001) (Fig. 2i). Finally, and in contrast to the findings of a previous semi-quantitative study [12], grey matter CD68 staining correlated closely with pTDP-43 severity in both sporadic and C9-ALS (Fig. 2k).

p62 is a ubiquitin-binding scaffold protein involved in the degradation of marked cargoes via selective autophagy, and is a known component of pTDP-43-immunoreactive inclusions. p62 was highest in the *OPTN* case. Among other genotypes, p62 was highest in *C9ORF72* cases, and lowest in *HNRNPA1* (Fig. 2b, e, h). p62 was significantly higher in sporadic than *FUS* (*p* = 0.0004) and *SOD1* (*p* = < 0.0001) cases but not *C9ORF72* disease (*p* = 0.92). We found a strong positive correlation between the extent of p62 and pTDP-43 in sporadic ALS [Pearson *r*(16) = 0.61, *p* = 0.006](Fig. 2j), however this relationship was not observed in C9-ALS to the same degree [Pearson *r*(14) = 0.53, *p* = 0.03](Fig. 2j), reflecting the existence of p62-positive, pTDP-43-negative dipeptide repeat proteins produced as a consequence of the *C9ORF72* expansion. In contrast to the pTDP-43-CD68 relationship identified in sporadic and C9-ALS, the extent of p62 aggregation did not correlate with CD68 in ALS-SOD1 and ALS-FUS (Fig. 2l).

Taken together, these results show that on average sporadic ALS is associated with a more pronounced motor cortex pTDP-43 proteinopathy and neuroinflammatory pathological phenotype than C9-ALS, implicate TDP-43 as a driver of microglial activation, and broadly highlight the significant variability in the severity of motor cortical pathology across the spectrum of ALS genotypes.

### Spatial and morphological distribution of motor cortical proteinopathy

**Fig. 3:**
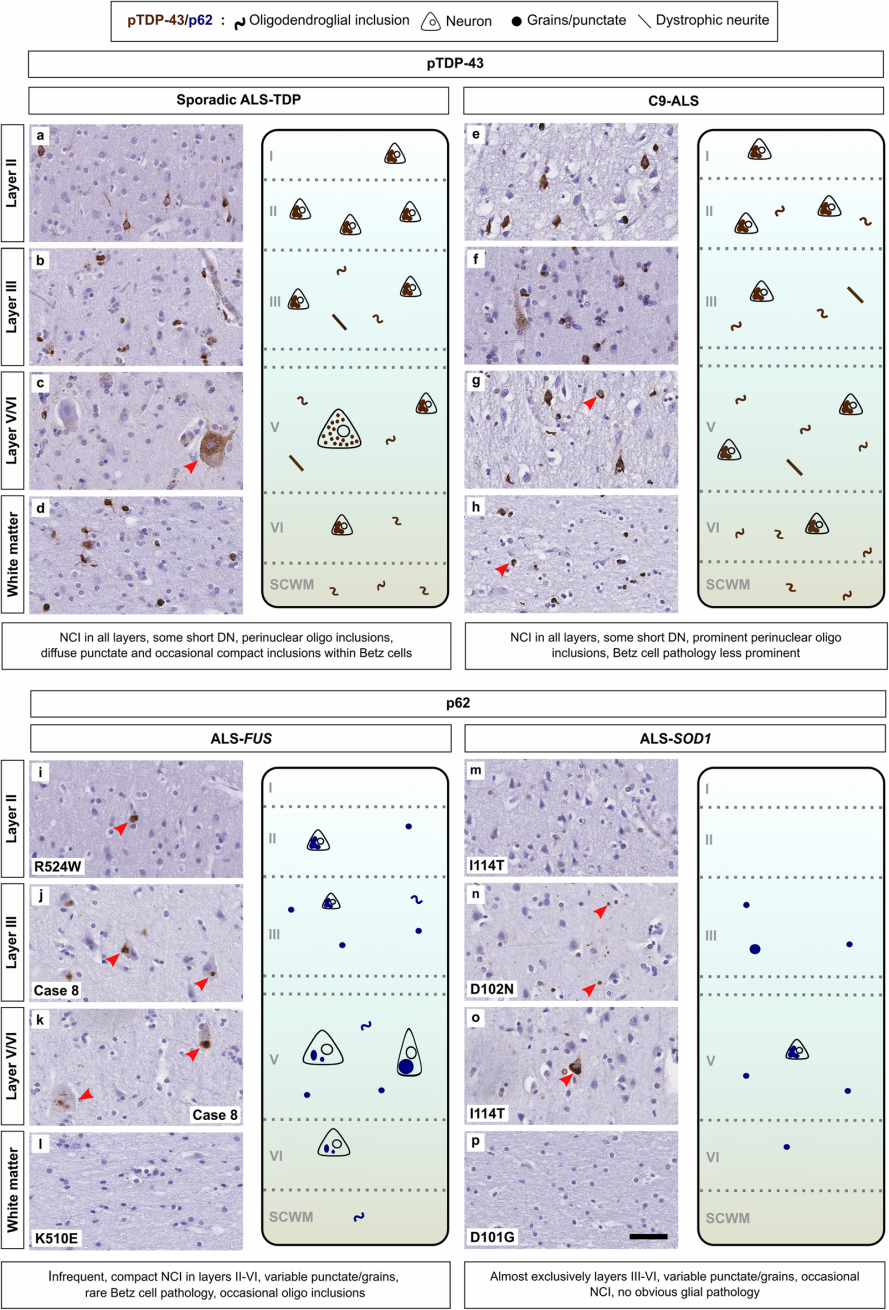
Spatial and morphological distribution of proteinopathy across genotypes in the ALS primary motor cortex. Sporadic ALS exhibits NCI and oligodendroglial pathology across layers I-VI as well as the subcortical white matter (**a**-**d**). The distribution pattern of pTDP-43 pathology was not entirely dissimilar between sporadic and C9-ALS (**e**-**f**), although there was a slight preponderance towards oligodendrocyte inclusions in C9 cases (Fig. 6g and h). *FUS* mutation cases demonstrated infrequent, compact p62-positive NCI with occasional granular inclusions, as well as occasional oligodendroglial pathology (i-l). *SOD1* mutation cases, by contrast, exhibited granular p62 staining confined to the middle cortical layers with infrequent NCI in layer V, without obvious glial pathology. Arrows highlight respective proteinopathy. Scale bar applicable to all panels = 50 μm

TDP-43 proteinopathy is the major neuropathological finding in sporadic and C9-ALS cases, while ALS-FUS and ALS-SOD1 cases are marked by p62-positive FUS and SOD1 aggregates, respectively [14, 51, 54]. Previous studies have described distribution patterns of cortical pTDP-43 pathology in sporadic ALS [77] and FTLD [47], but this has not been examined comparatively between genotypes in ALS. Given the significant variation in the extent of identified pathology between genotypes, we next qualitatively assessed the relative laminar distribution and morphologies of pTDP-43 and p62 proteinopathy in our s-IHC cohort (Fig. 3; for cases assessed see supplementary material).

While the extent was highly variable, the dominant pattern of cortical pTDP-43 pathology in sporadic cases consisted of granular, skein or compact neuronal aggregates, with occasional dot and short thread shaped dystrophic neurites, as well as ‘comma’ or ‘wisp’-like perinuclear oligodendroglial inclusions. Staining was mainly present within layers II-VI (Fig. 3a-c) but all ALS-TDP cases assessed exhibited at least one obvious example of oligodendroglial pTDP-43 pathology within the cortical/subcortical white matter, even in cases with the lowest average grey matter pTDP-43 overall (Fig. 3d). There was occasional, diffuse cytoplasmic punctate pathology within the giant pyramidal cells of Betz (Fig. 3c); compacted aggregates were rare. A harmonized subtyping system exists to describe the heterogeneity of pTDP-43 pathology in FTLD-TDP, based on the relative prevalence and distribution of dystrophic neurites (DN) and neuronal cytoplasmic inclusions (NCI). Assessment of primary motor cortex is not included in this system and it has been mostly applied to patients presenting initially with FTD. It is, however, recognised that cases of FTLD-ALS-TDP are most commonly associated with FTLD-TDP Type B neuropathology, both in sporadic as well as C9-FTLD-TDP cases [47]. Although motor cortex pTDP-43 morphology does not appear to be significantly different between FTLD-TDP and FTLD-ALS-TDP cases [78], for clarity, only C9-ALS cases with no clinical history of concomitant FTD were assessed here. Staining was similar in distribution to sporadic cases, with a slight preponderance towards oligodendroglial inclusions (Fig. 3e-h, Fig. 6g and h). However, Betz cell pathology was less prominent. No intranuclear pTDP-43-ir inclusions were seen in any of the sporadic or C9-ALS cases examined.

The cellular pattern of proteinopathy in our ALS-FUS and ALS-SOD1 cases was largely consistent with smaller case series described previously [31, 46]. ALS-FUS cases showed predominantly neuronal p62 aggregation within layers I-V in the form of sparse-moderate NCI and DN (Fig. 3i, j), although occasional oligodendroglial inclusions were seen. We found rare examples of p62-positive NCI within Betz cells in one of nine (case 8) ALS-FUS cases assessed (Fig. 3k). There were also occasional p62-positive aggregates within the subcortical white matter immediately below layer VI, but these did not reach the deeper white matter. By contrast, p62-positive aggregates in ALS-SOD1 cases were sparse, present almost exclusively within layers III-V, and consisted of variably sized granules (Fig. 3n) with occasional NCI (Fig. 3o). Notably, the majority of p62 aggregations appeared to be concentrated within the middle and deeper layers including layer V, which is the origin of corticospinal projections [50]. No obvious oligodendrocyte pathology was seen. This comparative assessment highlights the morphological variability in which genetic subtypes of ALS manifest as motor cortical disease.

### Sporadic ALS-TDP exhibits broad pathological homogeneity across the cortico-spinal neuraxis

**Fig. 4:**
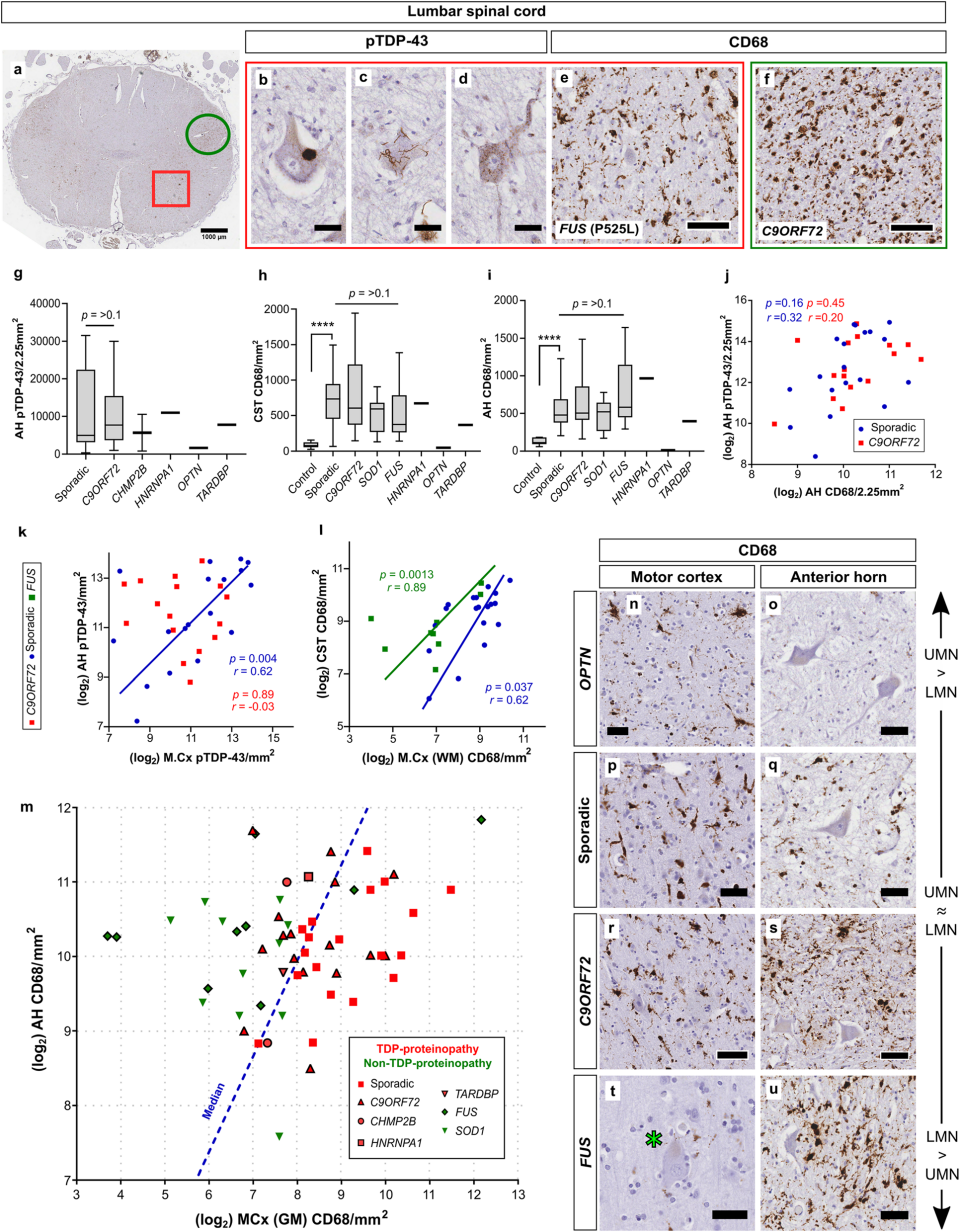
Sporadic ALS-TDP exhibits broad pathological homogeneity across the motor cortex-spinal cord neuraxis. CD68 and pTDP-43 expression was quantified within the anterior horn (**a**, red box) and corticospinal tract (a, green circle). The morphology of pTDP-43 pathology was varied in anterior horn lower motor neurons, including large discrete inclusions (**b**), thread-like skeins (**c**) and diffuse punctate (**d**). There was no significant difference in pTDP-43 severity between sporadic and *C9ORF72* cases (**g**). CD68 expression (**e**, **f**) was variable across ALS genotypes but was not statistically significant (**h**, **i**), and levels of pTDP-43 did not correlate with CD68 expression in the anterior horn (Pearson *r*; **j**). pTDP-43 in the motor cortex correlated with pTDP-43 in the anterior horn in sporadic disease but not C9-ALS (Pearson *r*; **k**), and CD68 correlated between the corticospinal white matter tract and the subcortical white matter in both *FUS* and sporadic cases (Pearson *r*; **l**) but not other genotypes (*p* > 0.2). CD68 in the lower anterior horn neuron was plotted against grey matter CD68 in the motor cortex (**m**). This was used to create a predominance ratio for each case that could be used as a covariate in subsequent models, where a lower ratio represents a tendency towards a burden of CD68 in the anterior horn. The dotted blue line in (**m**) represents the approximate median ratio of our cohort. Therefore, the further each case is from this line, the more extreme the relative UMN/LMN burden of CD68 the case exhibits. Cases are coloured according to whether they are TDP-proteinopathies (red) or not (green), showing that non-TDP-proteinopathies are more likely to exhibit a LMN neuropathological predominance when assessed via levels of activated microglia (ALS-*OPTN* case demonstrated extreme UMN predominance, but is excluded from graph for the clarity of other cases). Representative images of this variation across the neuraxis between genotypes, with an *OPTN* and *FUS* mutation representing upper and lower motor neuron spectral extremes, respectively (**n**-**u**). Asterisk in (**t**) highlights a seemingly normal Betz cell. AH, anterior horn; CST, corticospinal tract; UMN, upper motor neuron; LMN, lower motor neuron. Scale bars where not indicated (μm): b,c,d = 30, e,f,r,s = 75, all others = 50

As ALS patients commonly present clinically with a combination of upper and lower motor neuron signs and concomitant pathology, we next performed additional immunohistochemical staining on sections from the lumbar spinal cord region of each case in our single-stain IHC cohort to assess relative pathological predominance in upper or lower motor neuron compartments in our cohort. There was no significant difference in spinal cord pTDP-43 deposition between sporadic and *C9ORF72* cases (*p* = 0.65; Fig. 4g). The lateral corticospinal tract is composed of white matter axons that descend from the cortex and decussate at the level of the medullary pyramids, synapsing with lower motor neurons in the contralateral spinal cord. Although we found a trend for highest CD68 reactivity in the corticospinal tract of C9-ALS cases, this did not reach statistical significance compared with sporadic ALS-TDP cases (*p* = 0.91; Fig. 3h.) We also found no relationship between pTDP-43 and CD68 in the anterior horn (Fig. 4j).

Staining from the lumbar spinal cord from each case was next compared to its equivalent stain within the primary motor cortex to assess pathological relationships across the corticospinal neuraxis. Similar to a previous semi-quantitative study [11], pTDP-43 severity in the anterior horn correlated with that in the primary motor cortex in sporadic ALS-TDP cases [Pearson *r*(17) = 0.62, *p* = 0.004], however this relationship was not present in C9-ALS [Pearson *r*(14) = − 0.03, *p* = 0.89](Fig. 4k). CD68 staining within the lateral corticospinal tract correlated with CD68 staining in the primary motor cortex in sporadic [Pearson *r*(17) = 0.62, *p* = 0.0037] and *FUS* cases [Pearson *r*(7) = 0.89, *p* = 0.0013], but not other genotypes (*p* > 0.2)(Fig. 4l). Note that, with 3 out of 4 correlations being highly significant, simultaneous inference corrections do not modify the conclusions.

A previous semi-quantitative study found a correlation between microglial activation and neuronal loss in both the motor cortex and spinal cord [12]. We therefore next sought to assess the relative predominance of upper/lower motor neuron involvement in our cases by comparing the extent of pathology in the primary motor cortex to that in the anterior horn using CD68 as a surrogate marker of neurodegeneration. The extent of CD68 staining between motor cortex and spinal cord was expressed as a ratio, where smaller ratios represent a tendency towards a higher burden of CD68 in the anterior horn (Fig. 4m). Ratios were calculated as:

$$ \frac{M. Cx\  grey\ matter\  CD68/{mm^2}_{\left(\log \right)}}{Anterior\ horn\  CD68/{mm^2}_{\left(\log \right)}} $$

Many ALS-FUS and ALS-SOD1 cases exhibited cortical CD68 comparable to that of controls but showed significant staining within the spinal cord anterior horn (Fig. 4t,u), and 80% of all non-TDP-43 proteinopathies assessed fell below the calculated median predominance ratio of our cohort (Fig. 4m; Supp Figure 5). The single ALS-*OPTN* case demonstrated the most significant UMN predominance (Fig. 4n,o; Supp Figure 5). Together, these results show quantitatively that sporadic ALS-TDP exhibits high intraindividual pathological homogeneity across the cortico-spinal neuraxis, and that non-TDP-43 proteinopathies are more likely to display significant lower motor neuron predominance than TDP-43 disease, which is in broad concordance with clinical presentations commonly seen for these genotypes.

### GABAergic, GAD67+ interneurons only rarely accumulate pTDP-43 aggregates

**Fig. 5:**
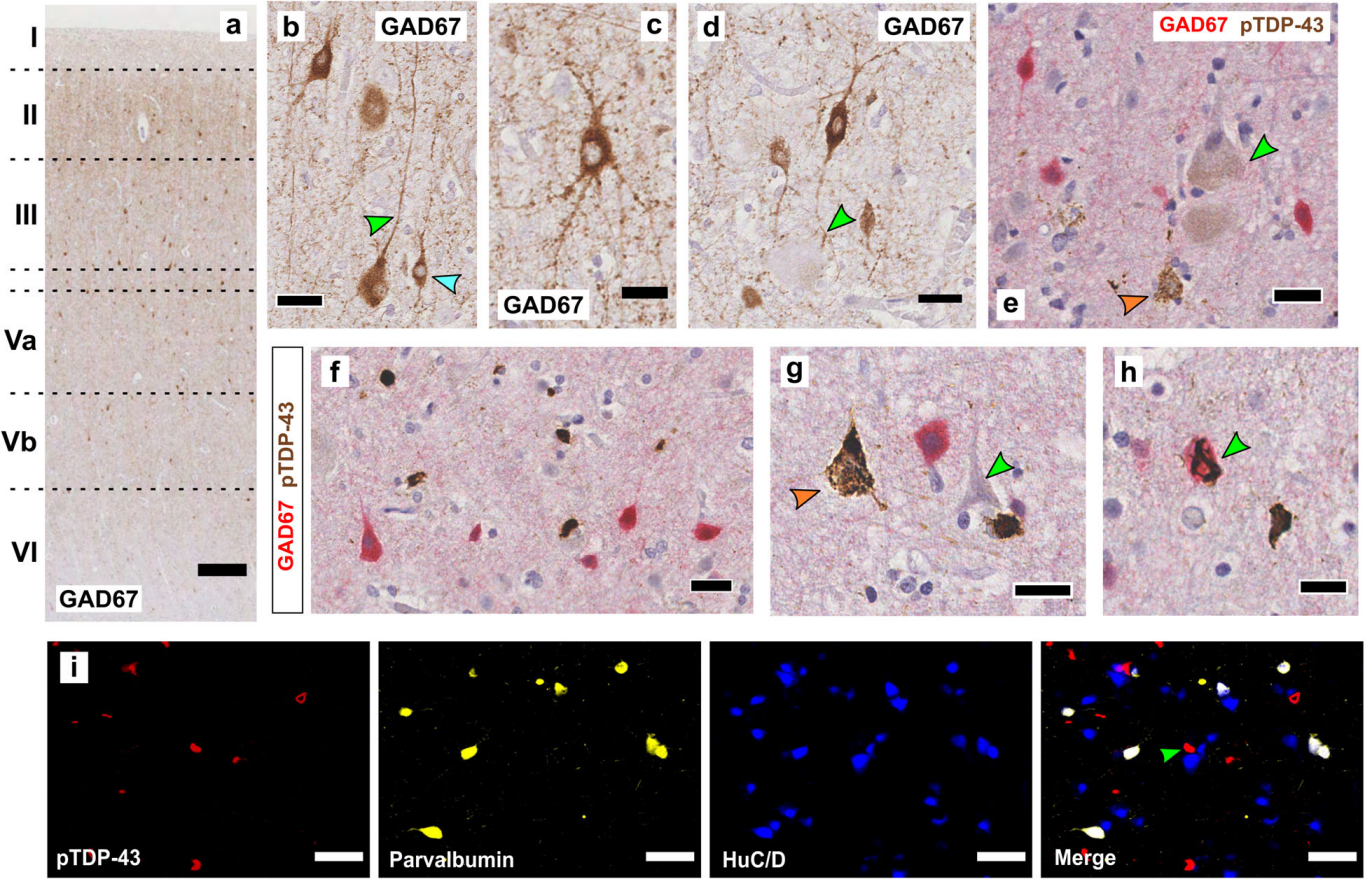
Cortical inhibitory interneurons rarely accumulate pTDP-43. Motor cortex GAD67+ neurons were present predominantly within layers II-V (**a**), and exhibited varied morphologies including large neurons with long apical dendrites (b, green arrow), smaller tufted neurons (**b**, blue arrow). GAD67 highlights multiple primary and secondary dendrites (**c**). GAD67 interneurons were seen synapsing directly on to Betz cell somata (**d**, green arrow). GAD67 neurons were devoid of granular or compact pTDP-43 aggregates, even in areas that otherwise contained typical ALS-TDP neuropathology (**e**-**g**; orange arrows: pTDP-43 in pyramidal cells, green arrow: lack of pTDP-43 in a Betz cell (5e) and non-Betz pyramidal cell (5 g)). We found only one instance of clear cytoplasmic colocalization of GAD67 and a compact pTDP-43 aggregate (5 h, green arrow). Multiplexed IHC revealed no evidence of parvalbumin colocalising with pTDP-43 (i, green arrow indicates HuD+, parvalbumin- neuron containing discrete pTDP-43 inclusion). Scale bars (μm): a = 300; b = 30; c,g = 20; d,e,f = 25; h = 15; i = 50

Gamma-aminobutyric acid (GABA) is the main inhibitory neurotransmitter in the brain. It is produced by glutamic acid decarboxylases (GADs) GAD65 and GAD67, of which GAD67 is generally constitutively active and produces > 90% of basal GABA levels [44]. GAD67 protein expression is a robust generic marker for cell bodies of inhibitory interneurons in human and mouse cortex [69, 72]. By contrast, no comparatively reliable tool is available as a generic marker for labelling of the soma of human excitatory, glutamatergic neurons, which include extratelencephalic pyramidal tract projection neurons. Therefore, we used GAD67-pTDP-43 double labelling to explore the vulnerability of GABAergic interneurons of the primary motor cortex to the accumulation of pTDP-43 aggregates (ALS *n* = 7, control *n* = 3; supplementary material), with the reasoning that this would allow us to obtain a histological window into potential imbalances of inhibitory and excitatory neurotransmission in ALS motor cortex. In controls, GAD67+ interneurons were mainly present in layers II-V (Fig. 5a), and included large neurons with long apical dendrites (Fig. 5b, green arrow) as well as smaller tufted neurons (Fig. 5b, blue arrow) as well as their dendritic protrusions (Fig. 5c). We also found examples of GAD67+ interneurons directly synapsing on to Betz cells soma (Fig. 5d, green arrow). Using GAD67-pTDP-43 double labelling in seven ALS cases assessed (see supplementary material for individual cases), we found that GAD67 neurons were devoid of granular or compact pTDP-43 aggregates, even in areas that otherwise contained typical ALS-TDP neuropathology (Fig. 5e-g). We found only one instance of clear cytoplasmic colocalization of GAD67 and a well-formed pTDP-43 aggregate (Fig. 5h), suggesting that pTDP-43 microscopic aggregates only rarely accumulate within interneurons. Additionally, we found no examples of compact pTDP-43 accumulation within Parvalbumin+ interneurons (Fig. 5j), further supporting the idea that pTDP-43 aggregation is more common within excitatory than inhibitory neurons in the ALS primary motor cortex.

### Cortical oligodendroglial, but not astroglial, pTDP-43 pathology is a defining feature of ALS-TDP

The main role of oligodendroglia is the support of neurons, either directly as cortical satellite glia or via the production of insulating myelin. As pTDP-43 accumulation is present within oligodendrocytes and their dysfunction has been implicated in pathogenesis of ALS [61], we sought to assess the numbers of oligodendrocytes within the primary motor cortex as well as the relative abundance of oligodendrocytes containing pTDP-43 inclusions in a subset of our cohort (ALS *n* = 7, control *n* = 3; see supplementary material for cases used).

**Fig. 6:**
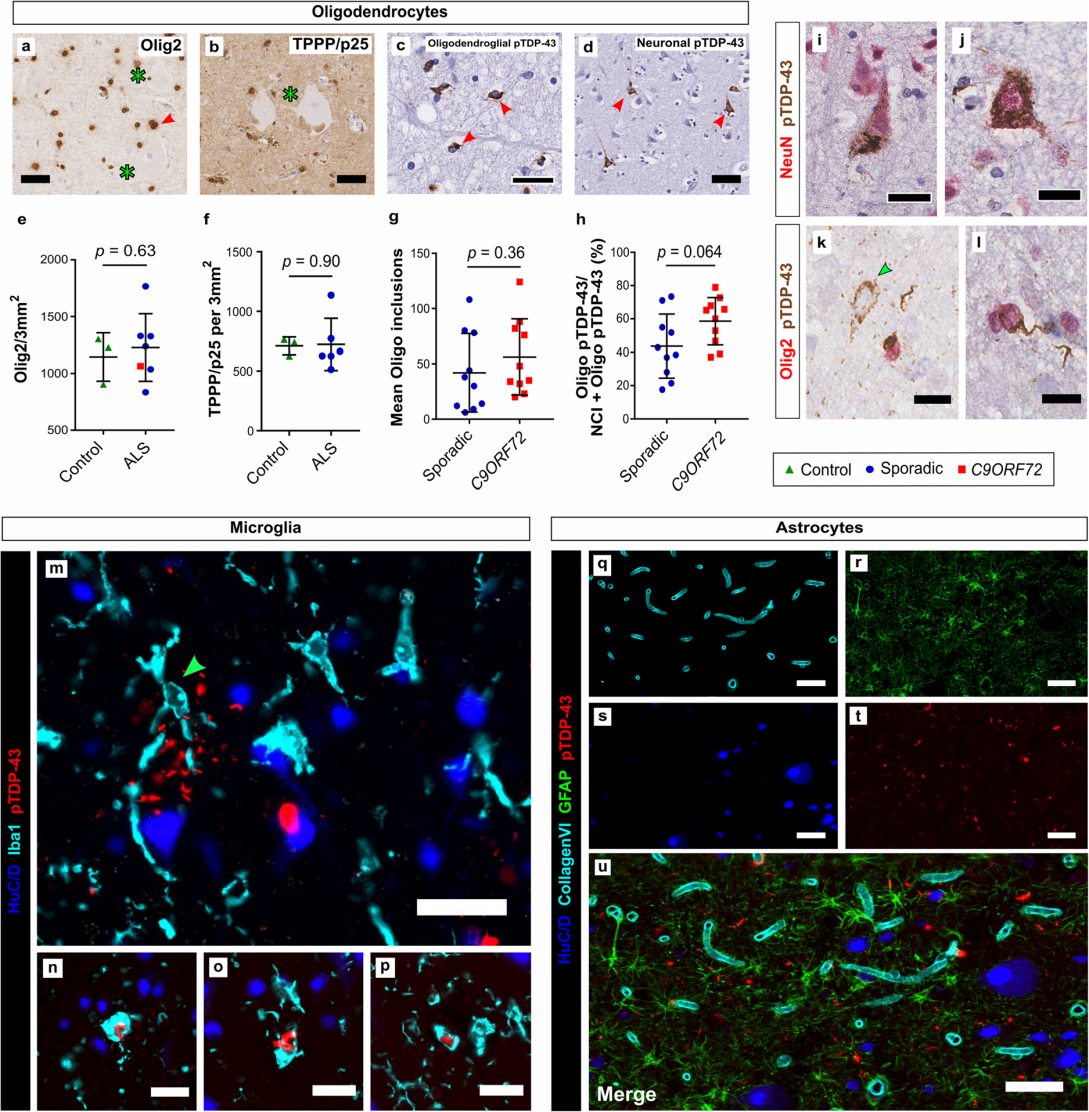
Differential vulnerability of glial cells in the ALS primary motor cortex. Olig2+ OPC (**a**) and mature TPPP/p25+ oligodendrocytes (**b**) were present in all layers of the primary motor cortex. Red arrow in (**a**) indicates satellite oligodendrocyte surrounding the apical dendrite of a Betz cell (green asterisks). There was no difference in the numbers of mature or immature oligodendrocytes between ALS and control cases (**e**, **f**). pTDP-43 inclusions are present in oligodendrocytes (**c**, red arrows indicated by characteristic ‘comma’ shaped inclusions) and neurons (**d**, red arrows indicate neuronal pTDP). Both the mean numbers of oligodendroglial inclusions and the relative predominance of pTDP-43 oligodendroglial pathology was higher in C9-ALS, but was not statistically higher than in sporadic disease (**g**, **h**). This relative distribution was confirmed qualitatively using double staining for pTDP-NeuN (**i**, **j**) and pTDP-Olig2 (**k**-**l**, green arrow indicates Olig2-negative neuronal inclusion). Multiplexed immunofluorescence reveals that Iba1+ microglia (**m**-**p**, green arrow in m indicates Iba1+ microglia extending processes to surround large layer V neuron containing pTDP-43 aggregates) occasionally contain pTDP-43, but we could find no clear evidence of this in GFAP+ astrocytes (**q**-**u**), even in cases with severe pTDP-43 pathology overall. Bars on graphs (**e**-**h**) represent means and SD. Scale bars (μm): c, i = 25, j = 20; k, l, m = 10; all others = 50

Olig2 is a transcription factor highly expressed in immature oligodendrocyte precursor cells (OPC)(Fig. 6a) while Tubulin polymerization-promoting protein (TPPP/p25) is highly expressed in mature oligodendrocytes (Fig. 6b) [10]. Similar to a previous study in the spinal cord [67], we found no significant difference in mature or immature oligodendrocyte numbers within the ALS-TDP motor cortex grey matter (Fig. 6e and f). We next assessed the prevalence of oligodendrocyte inclusions across our single-IHC cohort. We found at least one obvious instance of oligodendrocyte pTDP-43 inclusion in all ALS-TDP cases assessed (40/40), even in cases that exhibited very low levels of pTDP-43 pathology overall. We next blindly assessed the relative abundance of oligodendrocyte/neuronal pTDP-43 pathology in a randomised selection of ALS-TDP (*n* = 10) and C9-ALS cases (*n* = 10)(Supplementary material). Both the mean number of oligodendrocyte inclusions and the ratio of oligo/neuron inclusions was highest in C9-ALS but neither was significantly higher than in sporadic disease (Fig. 6g and h). We (OA) qualitatively reviewed all cases with respect to the topography and neuronal vs. oligodendroglial distribution of aggregates. We found that in human motor cortex FUS aggregates involve both neurons and oligodendroglia, but that SOD1 aggregates are restricted to neurons. The former assertion is in agreement with previous observations [5, 76], however, the latter is based on p62 staining, not staining with confirmation-specific antibody SEDI as this showed strong diffuse astroglial staining even in healthy controls (data not shown); nor did we have access to other conformation-specific SOD1 antibodies.

Microglia may phagocytose inclusions or cells that have been suitably marked for destruction as part of the innate immune system. As microglial activation correlates closely with the extent of pTDP-43 deposition (Fig. 2), we next analysed whether pTDP-43 inclusions could be visualised within microglia themselves. Similar to a previous study [59], we found several examples (Fig. 6m-p) of Iba1+ microglia either actively phagocytosing or containing pTDP-43 in several of the TDP-43 proteinopathy cases analysed. Astrocytes are a diverse glial subtype whose roles include mediation of potassium ions across the synapse and the provision of neurotrophic support. However while cortical astrogliosis is a feature of ALS [41], consistent with a previous study [77] there was no obvious perivascular pTDP-43 pathology suggestive of astrogliopathy and using pTDP-43-GFAP double labelling we were unable to find any clear evidence of pTDP-43 aggregates within GFAP+ astrocytes themselves (Fig. 6q-u).

Betz cells are giant pyramidal neurons unique to the primary motor cortex that reside within layer Vb, and can be distinguished histologically by their large size, accumulation of intracellular lipofuscin, and circumferential somatodendritic architecture. Similar to a previous study [9], we observed pTDP-43 accumulation within Betz cell perinuclear cytoplasm only rarely (supp. Figure 6a-d). However, in several cases we did find evidence of severe microgliosis surrounding or seemingly replacing large pyramidal neurons in layer V (supp. Figure 6e), suggesting that processes within these large neurons may induce a selective vulnerability to the microglial response, and indicating active neuronophagia even in the clinical end-stage of the disease (Fig. 2f), a phenomenon which has been previously reported in spinal motor neurons [58].

Together, these results highlight cortical pTDP-43 oligodendrocyte pathology as a defining feature of ALS-TDP across all studied genotypes, and suggest a clear neuronal/glial subtype specific vulnerability to proteoaggregation in the ALS primary motor cortex.

## Discussion

We report, to our knowledge, the first large-scale, digital microscopy-guided neuropathological analysis of the burden and pattern of proteinopathies of the primary motor cortex in ALS since the discovery of the main disease defining genotypes. A strength of our study is the combined use of image-analysis algorithms with statistical modelling to allow a comprehensive, fully quantitative approach to pathological assessment. We consider these quantitative traits to be more proximally related to genetic variables and pathobiology than clinical data (e.g. UMN vs. LMN predominance), which were limited, not quantitative and unlikely to be reflective of neuraxis involvement at the end stage of the disease. Importantly, the genetic stratification of our cohort allowed us to begin to assess ALS neuropathological endophenotypes, the utility of which has been demonstrated in other settings, such as frontotemporal lobar degeneration and hippocampal sclerosis [2, 6, 26, 27, 53].

Our dataset stems from an independent, previously unpublished cohort of individuals with ALS stratified by genotype. It allows us to contribute several important findings to the field: First, in the group of ALS associated with phosphorylated (p)TDP-43 proteinopathy, sporadic disease was characterised by a higher pTDP burden in the motor cortex than C9-ALS, and all genotypes in this group demonstrated oligodendroglial pTDP-43 inclusion body pathology. Second, overall motor cortex proteinopathy burden of ALS-FUS and ALS-SOD1 is less severe than that of ALS-TDP. Third, inhibitory interneurons appear to be less prone to accumulation of microscopic pTDP-43 aggregates than excitatory neurons.

A key point in discussions concerning selective vulnerability is the distinction between cellular vulnerability to proteinopathy, and vulnerability to degeneration itself. The toxicity of TDP-43 has been demonstrated in vitro through a range of mechanisms including its effects on endocytosis, autophagy and stress-granule formation [34, 37, 45]. However, the cellular effect of this toxicity in humans is less well understood, and the relative contribution of toxic gain of function and loss of physiological nuclear function of TDP-43 remains difficult to disentangle. We observed high interindividual variation in the extent of pTDP-43 across our cohort, including in patients of the same genotype. pTDP-43 aggregation was amongst the lowest in the single *TARDBP* mutation case assessed (Fig. 2a,g; supp Figure 2), however notably it has not been formally established that the pattern and/or severity of pTDP-43 pathology in *TARDBP* mutation cases is the same as in ALS-TDP or C9-ALS. Understanding the extent of this variation across genotypes is relevant because clearance of pathological cytoplasmic TDP-43 rescues motor deficits and extends lifespan in mice [82], and clinical trials involving immunotherapy are ongoing in humans. Unidentified genetic factors are likely an influence on the overall extent of pathology, however the majority of cases used in this study were sequenced using whole-exome or SNP-array based sequencing using blood-derived DNA, meaning any potential CNS-specific variation in the majority of the genome is unaccounted for. In our study, the main genotypes were sporadic disease and C9-ALS; we did not have data on *ATXN2* intermediate expansions. In this context it is interesting to note that Yang et al. [85], using immunoblots for pTDP-43, found evidence of higher levels of pTDP-43 in motor cortex of C9-ALS and in spinal cord of ALS-TDP with intermediate *ATXN2* expansions. To our knowledge, it has not been formally studied how increased levels of biochemically-determined insoluble pTDP-43 relate to unbiased quantification of light-microscopically visible pTDP-43 aggregates in the same anatomical compartment. Our data revealed a significant, but not dramatic difference in microscopic pTDP-43 burden in motor cortex of C9-ALS carriers versus sporadic ALS-TDP (*p* = 0.043); however, we observed a trend towards higher oligodendroglial pTDP-43 burden in C9-ALS.

We detected a differential vulnerability of specific cell types to the aggregation of pTDP-43. This can be summarised as follows: excitatory neurons within the motor cortex are more prone to form pTDP-43 aggregates than inhibitory interneurons, and oligodendroglial, but not astroglial, pTDP-43 pathology should be considered a defining feature of ALS-TDP in cases with and without proven genetic aetiology. We found evidence of oligodendrocyte pathology in every ALS-TDP case assessed in this study, demonstrating cortical oligodendroglial proteinopathy as a defining feature of the disease. However, the mechanisms by which oligodendroglial aggregations potentially cause dysfunction are unclear. While the cellular origin of TDP-43 pathology in humans is not known, studies have suggested that TDP-43 can be transmitted between cells both in culture and in vivo [20, 63]. It is plausible therefore that pTDP-43 oligodendroglial pathology is preceded by cell-to-cell transmission of seed-competent soluble TDP-43, propagating its non-cell autonomous effects. Notably, and contrasting observations in the spinal cord [21], we found that this pattern of cortical neuronal and oligodendroglial proteinopathy is also a hallmark of ALS-FUS motor cortex, but not of ALS-SOD1. This is unexpected, as the concept of non-neuronal - including oligodendroglial - contributions to ALS neurodegeneration has been established in the *SOD1*^G93A^ mouse model [36, 61]. Whether this indicates a differential vulnerability of oligodendroglia to RNA-binding protein homeostasis or of different oligodendroglial subtypes remains to be established.

There is no doubt however that microglial activation occurs across all genetic subtypes of human ALS, although whether this response is in itself pathogenic or primarily a subsequent reactive event is debated. Our results show that the extent of this response in the primary motor cortex varies significantly between ALS genotypes, and confirms findings ante mortem concerning the difference in microglial burden between upper and lower motor neurons in sporadic ALS [74, 80]. Several ALS genes have been implicated in the innate immune response specifically; for example *C9ORF72* is highly expressed in myeloid cells [57, 66], conditional myeloid deletion of *OPTN* results in dysmyelination and axonopathy [32], and removal of mutant SOD1 from microglia prolongs lifespan in transgenic mice [7]. However, comparably low numbers of cortical activated microglia were seen in *SOD1* and *FUS* cases, which exhibited a relatively low extent of cortical proteinopathy, while the highest microglial response was seen in sporadic and *C9ORF72* cases, which on average demonstrated a higher burden of concomitant pTDP-43. This suggests that TDP-43 proteoaggregation specifically determines the extent of the microglial response, rather than the expression of a mutant protein within microglia themselves. We found several examples of pTDP-43 aggregates within microglia (Fig. 6), though we cannot say whether these cells actively phagocytosed the aggregates, or the inclusions arose within the microglia as result of the disease process. We also found no evidence of pTDP-43 inclusions within GFAP+ astroglia, despite cortical astrogliosis being a feature in ALS [41]. Similar to a previous study, we also found only some evidence of compact pTDP-43 aggregates within Betz cell somata [9], although we did find evidence of severe microgliosis and neuronophagia surrounding large layer Vb neurons in several cases, which has also been previously reported [33]. It has been noted that some ALS Betz cells seem to lose expression of the native protein entirely [9]. This raises interesting questions regarding whether Betz cells are perhaps selectively vulnerable to the toxicity conferred by soluble TDP-43 and its effects on microglia, and, relative to other neurons, whether they lack the capacity to concentrate insoluble TDP-43 in microscopically visible compact inclusions, which may be neuroprotective. In this context it is interesting to note that our data suggest inhibitory interneurons of the primary motor cortex do not seem to form abundant microscopically visible compact pTDP-43 aggregates either. We may, therefore, infer that non-Betz excitatory neurons carry the vast majority of neuronal pTDP-43 aggregate burden in human motor cortex. Whether this reflects cell-intrinsic biochemical traits of GABAergic neurons or is an expression of an inhibitory-excitatory local network imbalance remains unknown. Experimental evidence appears to indicate that excitatory layer V pyramidal activity is directly associated with the accumulation of ubiquitinated aggregates in TDP-43 mutant mice [87], however it is likely that more complex genotype-dependent interactions between neurons are at play [38]. In any case, our inference that excitatory pyramidal neurons, but not inhibitory interneurons, are the primary neuronal cell type accumulating pTDP-43 aggregates is consistent with the hypothesis that pTDP-43 propagation through the human nervous system is initiated by them [8].

Our study contains several limitations. We analysed a region of the primary motor cortex corresponding to the hand region of the homunculus wherever possible, however we cannot guarantee that this region was analysed in every case, and it is possible that the expression of proteins described in this paper vary across the somatotopically defined regions of the primary motor cortex. Additionally, we have not stratified our cohort by hemisphere sampled, and it is possible that ALS pathology varies between the same region of different hemispheres, particularly as initial clinical presentation is often lateralised. We note that there is some evidence of asymmetrical TDP-43 pathology in FTLD (without ALS) and Alzheimer’s disease [75], but such asymmetry was not found in two cases of ALS-FTLD [39]. The precise variation of ALS pathology across hemispheres is therefore of an unknown significance. We also used CD68 to create a predominance ratio for each case that reflected the comparable levels of involvement between the primary motor cortex and lumbar spinal cord. This was necessary because there is a degree of corticospinal motor neuron loss even in patients that display almost purely LMN signs [70]. Ideally, neuronal loss itself would have been used to create a predominance indicator. However, NeuN, the most commonly used pan-neuronal marker in mouse studies, displays a strong fixation dependency and is unsuitable for use with the kind of archival material used in the single-IHC component of this study, in which we aimed to achieve the highest possible numbers of cases for analysis.

In summary, we used a quantitative immunohistochemical approach to comprehensively analyse pathology within the primary motor cortex in a large cohort of genetically-defined ALS cases. Our results demonstrate a clear genotype-specific vulnerability to ALS proteinopathy, which may provide targets for the design of future therapeutics through genotype-specific amelioration of cortical pathology.

## Supplementary information

**Additional file 1 Supp Fig. 1:** Additional staining of pathology in the motor cortex and spinal cord. Staining for OPTN was highly positive in all neurons in controls (A), but absent in an *OPTN* mutation case (D), reflecting the truncation of the protein upstream of the antibody epitope. Asterisks in (B) mark the presence of Betz cells. Arrows in (E) mark mislocalized and aggregated FUS inclusions. Arrows in (F) mark the presence of aggregated SOD1 protein in a case with the *SOD1* D101G mutation (case 31). As discussed in the text, antibody SOD1 SPC-206 did highlight solid compact and skein aggregates but had strong background staining (C), making it unsuitable for quantitative automated image analysis. P62 was used instead as a marker for compact SOD-1 associated protein aggregates; granular aggregation of misfolded wild-type SOD1, which has been suggested to be present in all genotypes of ALS, was not revealed by p62 immunohistochemistry [22]. Scale bar applicable to all panels = 50 μm. **Supp Fig. 2:** Significant variation in the extent of pTDP-43 pathology between *TARDBP* and *OPTN* ALS mutation cases. pTDP-43 pathology in the *TARDBP* case was sparse, and consisted almost exclusively of compact NCI (a,b, red arrows highlight pathology). In contrast, pathology was severe and widespread in the homozygous ALS-OPTN case (c,d), with NCI, oligo inclusions and dystrophic neurites in all layers, including the subcortical white matter (e). **Supp Fig. 3:** CD68 staining between the primary motor cortex (a) and lumbar spinal cord (b) white and grey matter is positively correlated in all the genotypes tested; Pearson r, results as on figure. Best fit lines are manually added for illustrative purposes. **Supp Fig. 4:** Assessment of anterior horn neuron size. Anterior horn degeneration and shrinkage was most prominent in *FUS* (b) and *SOD1* cases, and noticeably less severe in the single ALS-*OPTN* case (c), however there was significant intraindividual differences within genotypes (d). pTDP-43 aggregation in the anterior horn did not correlate with anterior horn neuron shrinkage/loss in either sporadic (blue dots) or *C9ORF72* disease (red squares) (e). **Supp Fig. 5:** Calculated predominance ratios for single-IHC cohort using CD68 as a surrogate marker of neurodegeneration. Ratios were calculated by dividing the log expression of motor cortex CD68/mm^2^ by the log expression of anterior horn CD68/mm^2^. Lower ratios therefore represent a higher LMN burden of activated microglia. **Supp Fig. 6:** Betz cells occasionally display pTDP-43 aggregation but also occasionally nodular microgliosis and neuronophagia. MxIF analysis did not reveal significant evidence of pTDP-43 within Betz cells (a,b, green lined arrows indicate unaffected Betz cells), but it can occasionally be seen in some cases (c,d). However we did also find evidence of nodular microgliosis surrounding large layer V neurons in some cases (e). UL = upper layers, DL = deeper layers. Scale bars where not indicated (μm): a = 40, b,e = 50. **Supp** Table 3**:** Numerical results for Olig and TPPP/p25 quantification.

**Additional file 2.**

## Abbreviations

ALS: Amyotrophic Lateral Sclerosis
FTD: Frontotemporal Dementia
FTLD: Frontotemporal lobar degeneration
TDP-43: Transactive Response DNA binding protein 43-kDa
*FUS*: Fused in Sarcoma
*OPTN*: Optineurin
LMN: Lower motor neuron
UMN: Upper motor neuron
MxIF: Multiplexed immunofluorescence
*SOD1*: Superoxide dismutase 1
*C9ORF72*: Chromosome 9 open reading frame 72
