# Supplementary material for: Quantitative patterns of motor cortex proteinopathy across ALS genotypes
Source: Acta Neuropathol Commun. 2020 Jul 2;8:98. doi: 10.1186/s40478-020-00961-2 (PMC7331195; doi:10.1186/s40478-020-00961-2)
Supplement: Supplementary file 1 — Additional file 1 Supp Fig. 1: Additional staining of pathology in the motor cortex and spinal cord. Staining for OPTN was highly positive in all neurons in controls (A), but absent in an OPTN mutation case (D), reflecting the truncation of the protein upstream of the antibody epitope. Asterisks in (B) mark the presence of Betz cells. Arrows in (E) mark mislocalized and aggregated FUS inclusions. Arrows in (F) mark the presence of aggregated SOD1 protein in a case with the SOD1 D101G mutation (case 31). As discussed in the text, antibody SOD1 SPC-206 did highlight solid compact and skein aggregates but had strong background staining (C), making it unsuitable for quantitative automated image analysis. P62 was used instead as a marker for compact SOD-1 associated protein aggregates; granular aggregation of misfolded wild-type SOD1, which has been suggested to be present in all genotypes of ALS, was not revealed by p62 immunohistochemistry [22]. Scale bar applicable to all panels = 50 μm. Supp Fig. 2: Significant variation in the extent of pTDP-43 pathology between TARDBP and OPTN ALS mutation cases. pTDP-43 pathology in the TARDBP case was sparse, and consisted almost exclusively of compact NCI (a,b, red arrows highlight pathology). In contrast, pathology was severe and widespread in the homozygous ALS-OPTN case (c,d), with NCI, oligo inclusions and dystrophic neurites in all layers, including the subcortical white matter (e). Supp Fig. 3: CD68 staining between the primary motor cortex (a) and lumbar spinal cord (b) white and grey matter is positively correlated in all the genotypes tested; Pearson r, results as on figure. Best fit lines are manually added for illustrative purposes. Supp Fig. 4: Assessment of anterior horn neuron size. Anterior horn degeneration and shrinkage was most prominent in FUS (b) and SOD1 cases, and noticeably less severe in the single ALS-OPTN case (c), however there was significant intraindividual differences within genotypes (d). pTDP-43 a [file 40478_2020_961_MOESM1_ESM.pdf]

Supplementary material

Quantification algorithm parameters

All quantification conducted using QuPath software, algorithms in Apache groovy.

p62 positive pixel count

```
setImageType('BRIGHTFIELD_H_DAB');
setColorDeconvolutionStains('{"Name" : "H-DAB default", "Stain 1" : "Hematoxylin",
"Values 1" : "0.65111 0.70119 0.29049 ", "Stain 2" : "DAB", "Values 2" : "0.26917
0.56824 0.77759 ", "Background" : " 255 255 255 "}');
runPlugin('qupath.imagej.detect.tissue.PositivePixelCounterIJ', '{"downsampleFactor": 3,
"gaussianSigmaMicrons": 2.0,  "thresholdStain1": 0.3,  "thresholdStain2": 0.2,
"addSummaryMeasurements": true}');
```

pTDP-43 positive pixel count

```
setImageType('BRIGHTFIELD_H_DAB');
setColorDeconvolutionStains('{"Name" : "H-DAB default", "Stain 1" : "Hematoxylin",
"Values 1" : "0.65111 0.70119 0.29049 ", "Stain 2" : "DAB", "Values 2" : "0.26917
0.56824 0.77759 ", "Background" : " 255 255 255 "}');
runPlugin('qupath.imagej.detect.tissue.PositivePixelCounterIJ', '{"downsampleFactor": 2,
"gaussianSigmaMicrons": 2.0,  "thresholdStain1": 0.2,  "thresholdStain2": 0.3,
"addSummaryMeasurements": true}');
```

CD68 cell count

```
setImageType('BRIGHTFIELD_H_DAB');
setColorDeconvolutionStains('{"Name" : "H-DAB default", "Stain 1" : "Hematoxylin",
"Values 1" : "0.65111 0.70119 0.29049 ", "Stain 2" : "DAB", "Values 2" : "0.26917
0.56824 0.77759 ", "Background" : " 255 255 255 "}');
runPlugin('qupath.imagej.detect.nuclei.PositiveCellDetection',
'{"detectionImageBrightfield": "Optical density sum",  "requestedPixelSizeMicrons": 1.0,
"backgroundRadiusMicrons": 8.0,  "medianRadiusMicrons": 0.0,  "sigmaMicrons": 3.0,
"minAreaMicrons": 10.0,  "maxAreaMicrons": 400.0,  "threshold": 0.2,  "maxBackground":
2.0,  "watershedPostProcess": true,  "excludeDAB": false,  "cellExpansionMicrons": 5.0,
"includeNuclei": true,  "smoothBoundaries": true,  "makeMeasurements": true,
"thresholdCompartment": "Cell: DAB OD mean",  "thresholdPositive1": 0.15,
"thresholdPositive2": 0.4,  "thresholdPositive3": 0.6,  "singleThreshold": true}');
```

OLIG2 cell count

```
setImageType('BRIGHTFIELD_H_DAB');
setColorDeconvolutionStains('{"Name" : "H-DAB default", "Stain 1" : "Hematoxylin",
"Values 1" : "0.65111 0.70119 0.29049 ", "Stain 2" : "DAB", "Values 2" : "0.26917
0.56824 0.77759 ", "Background" : " 255 255 255 "}');
runPlugin('qupath.imagej.detect.nuclei.PositiveCellDetection',
'{"detectionImageBrightfield": "Optical density sum",  "requestedPixelSizeMicrons": 0.5,
"backgroundRadiusMicrons": 8.0,  "medianRadiusMicrons": 0.0,  "sigmaMicrons": 1.5,
"minAreaMicrons": 10.0,  "maxAreaMicrons": 400.0,  "threshold": 0.5,  "maxBackground":
2.0,  "watershedPostProcess": true,  "excludeDAB": false,  "cellExpansionMicrons": 5.0,
"includeNuclei": true,  "smoothBoundaries": true,  "makeMeasurements": true,
"thresholdCompartment": "Nucleus: DAB OD mean",  "thresholdPositive1": 0.2,
"thresholdPositive2": 0.4,  "thresholdPositive3": 0.6,  "singleThreshold": true}');
```

TPPP/p25 cell count

```
setImageType('BRIGHTFIELD_H_DAB');
setColorDeconvolutionStains('{"Name" : "H-DAB default", "Stain 1" : "Hematoxylin",
"Values 1" : "0.65111 0.70119 0.29049 ", "Stain 2" : "DAB", "Values 2" : "0.26917
0.56824 0.77759 ", "Background" : " 255 255 255 "');
runPlugin('qupath.imagej.detect.nuclei.PositiveCellDetection',
'{"detectionImageBrightfield": "Optical density sum",  "requestedPixelSizeMicrons": 0.5,
"backgroundRadiusMicrons": 8.0,  "medianRadiusMicrons": 0.0,  "sigmaMicrons": 2.0,
"minAreaMicrons": 10.0,  "maxAreaMicrons": 400.0,  "threshold": 0.55,  "maxBackground":
2.0,  "watershedPostProcess": true,  "excludeDAB": false,  "cellExpansionMicrons": 5.0,
"includeNuclei": true,  "smoothBoundaries": true,  "makeMeasurements": true,
"thresholdCompartment": "Nucleus: DAB OD mean",  "thresholdPositive1": 0.2,
"thresholdPositive2": 0.4,  "thresholdPositive3": 0.6,  "singleThreshold": true}');
```

Supp fig 1

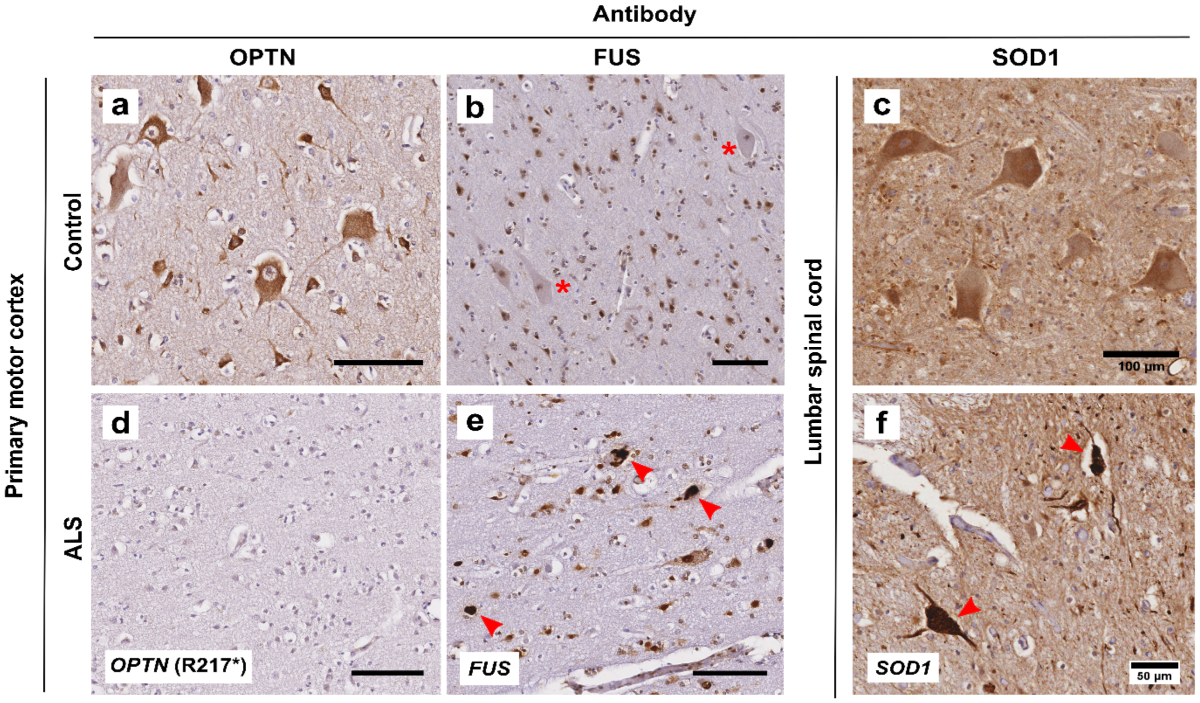

Antibody
OPTN
FUS
SOD1
Primary motor cortex
Control
ALS
Lumbar spinal cord
a
b
c
d
e
f
100 µm
OPTN (R217*)
FUS
SOD1
50 µm

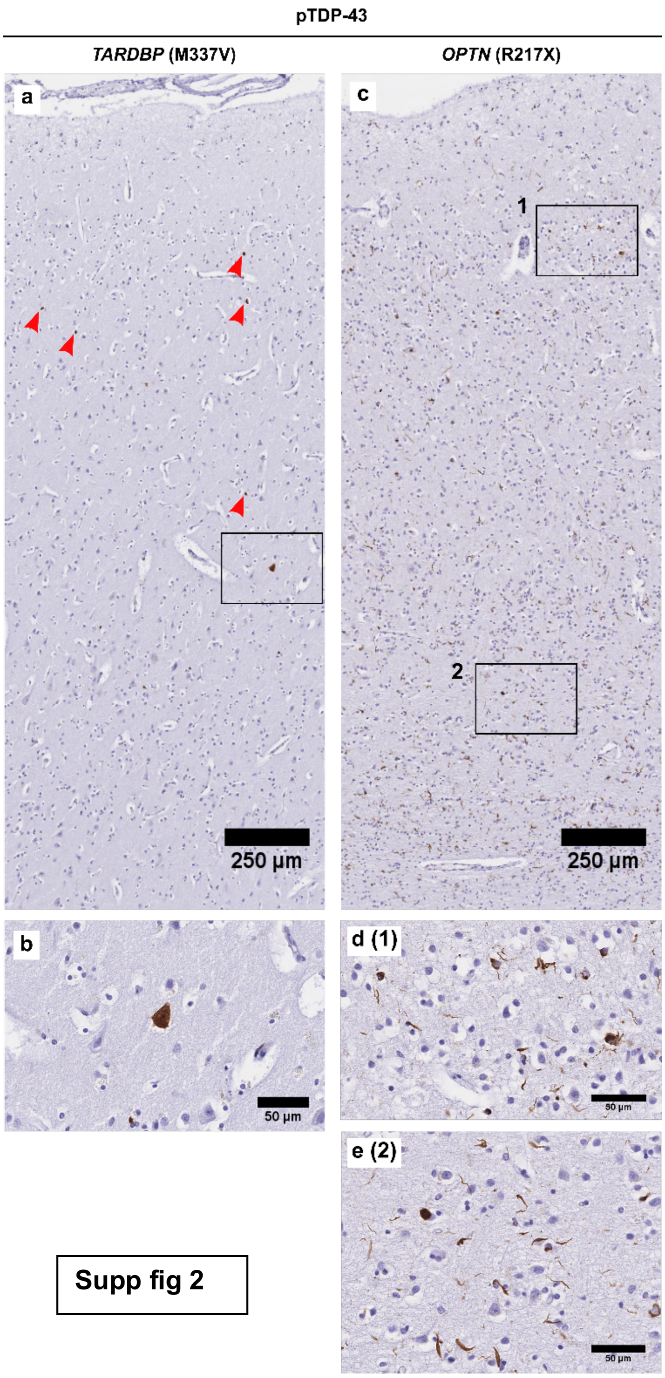
pTDP-43
TARDBP (M337V)
OPTN (R217X)
a
c
1
2
250 µm
250 µm
b
50 µm
d (1)
50 µm
e (2)
50 µm

Supp fig 2

Supp fig 3

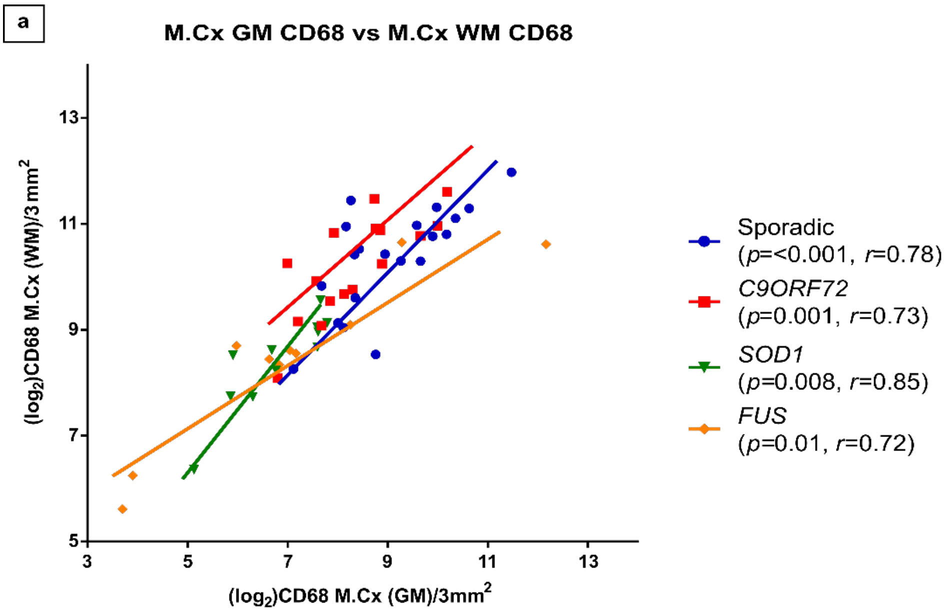
a
M.Cx GM CD68 vs M.Cx WM CD68
(log2)CD68 M.Cx (WM)/3mm2
(log2)CD68 M.Cx (GM)/3mm2
13
11
9
7
5
3
5
7
9
11
13
Sporadic (p=<0.001, r=0.78)
C9ORF72 (p=0.001, r=0.73)
SOD1 (p=0.008, r=0.85)
FUS (p=0.01, r=0.72)

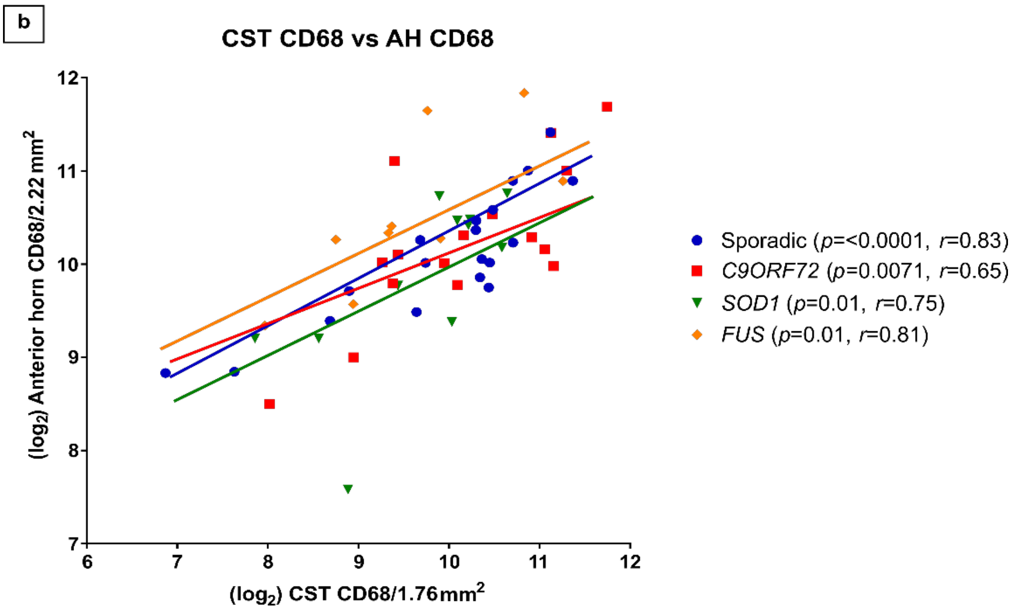
b
CST CD68 vs AH CD68
(log2) Anterior horn CD68/2.22 mm2
(log2) CST CD68/1.76mm2
12
11
10
9
8
7
6
7
8
9
10
11
12
Sporadic (p=<0.0001, r=0.83)
C9ORF72 (p=0.0071, r=0.65)
SOD1 (p=0.01, r=0.75)
FUS (p=0.01, r=0.81)

Supp fig 4

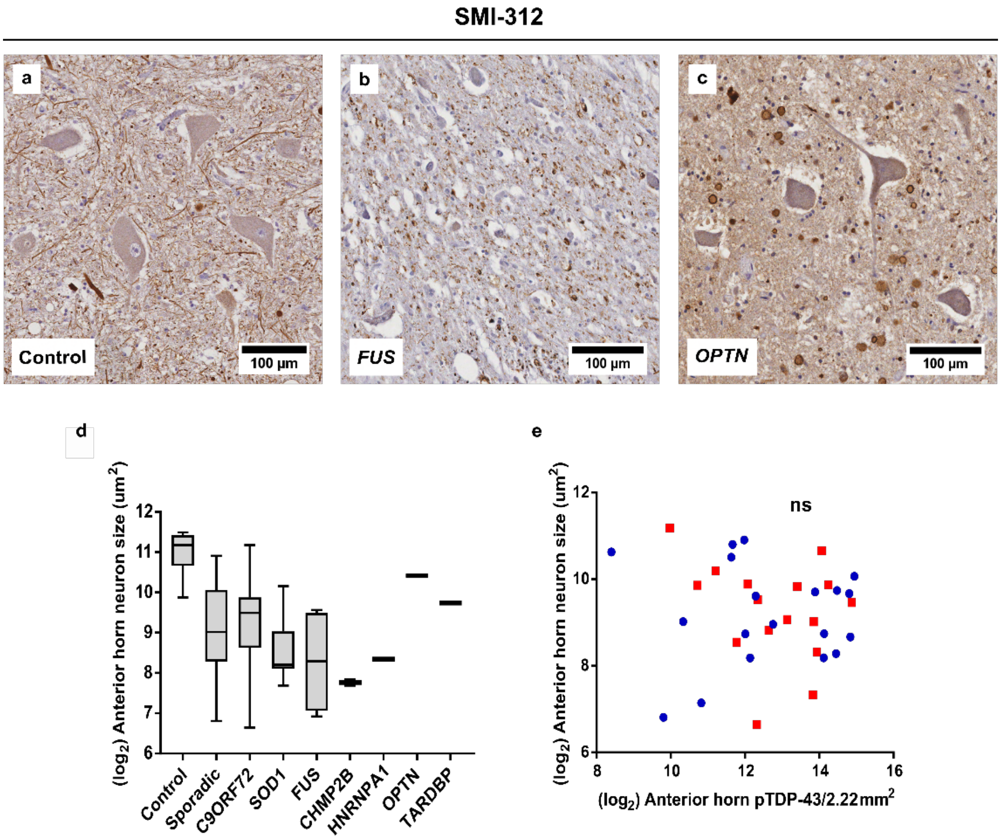

SMI-312
a
Control
100 µm
b
FUS
100 µm
c
OPTN
100 µm
d
(log2) Anterior horn neuron size (um2)
12
11
10
9
8
7
6
Control
Sporadic
C9ORF72
SOD1
FUS
CHMP2B
HNRNPA1
OPTN
TARDBP
e
(log2) Anterior horn neuron size (um2)
ns
12
10
8
6
8
10
12
14
16
(log2) Anterior horn pTDP-43/2.22mm2

Supp fig 5

Percentile	Genotype	Predominance ratio
	OPTN	2.397
	Sporadic	1.015
	Sporadic	1.004
	FUS	0.991
	Sporadic	0.990
	Sporadic	0.959
	C9ORF72	0.949
	Sporadic	0.938
	SOD1	0.935
	Sporadic	0.932
	C9ORF72	0.914
80%	*C9ORF72*	0.910
	Sporadic	0.880
	C9ORF72	0.864
	Sporadic	0.861
	Sporadic	0.852
	C9ORF72	0.847
	Sporadic	0.829
	Sporadic	0.811
	C9ORF72	0.794
	FUS	0.791
	Sporadic	0.787
	Sporadic	0.779
	C9ORF72	0.758
	SOD1	0.755
	Sporadic	0.747
	CHMP2B	0.747
	Sporadic	0.740
	C9ORF72	0.738
	Sporadic	0.733
Median	Sporadic	0.725
	Sporadic	0.721
	C9ORF72	0.718
	Sporadic	0.709
	TARDBP	0.706
	C9ORF72	0.699
	C9ORF72	0.685
	HNRNPA1	0.672
	SOD1	0.670
	C9ORF72	0.667
	SOD1	0.666
	C9ORF72	0.664
	C9ORF72	0.639
	SOD1	0.634
	C9ORF72	0.628
	CHMP2B	0.627
	SOD1	0.627
	SOD1	0.602
20%	*FUS*	0.567
	FUS	0.549
	FUS	0.522
	FUS	0.520
	SOD1	0.519
	C9ORF72	0.513
	SOD1	0.507
	SOD1	0.451
	SOD1	0.380
	FUS	0.271
	FUS	0.255
	FUS	0.232

UMN

LMN

Supp fig 6

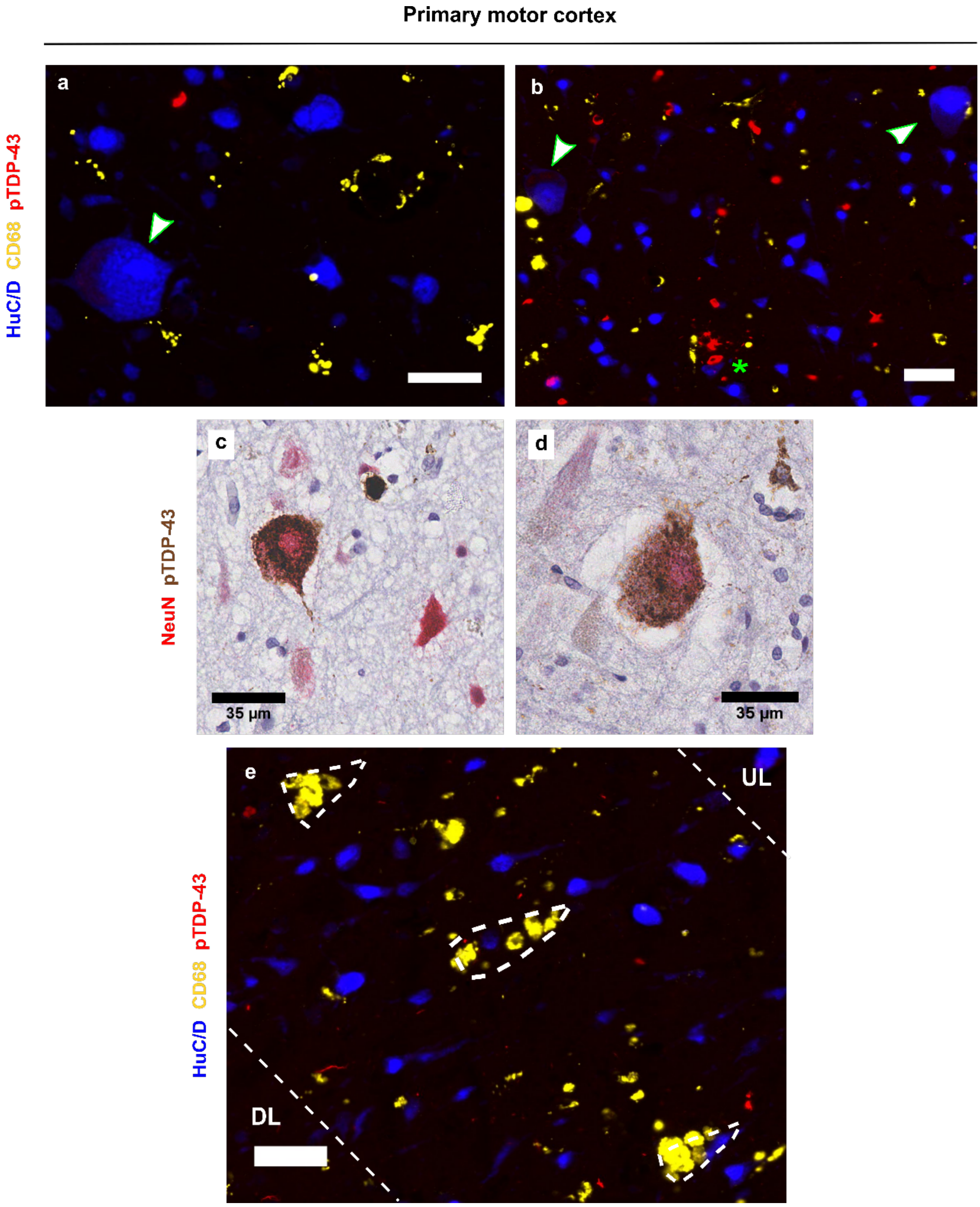
Primary motor cortex
a
b
HuC/D CD68 pTDP-43
c
d
NeuN pTDP-43
35 µm
35 µm
e
UL
DL
HuC/D CD68 pTDP-43

Genotype	Mean OLIG2+ oligodendrocytes/3mm^2	Mean Tppp/p25+ oligodendrocytes/3mm^2
Control	1229	741
Control	1303	626
Control	903	766
sALS	1766	1135
sALS	1329	627
sALS	1326	666
sALS	1036	626
sALS	835	513
sALS	1237	773
C9-ALS	1064	Not available

Supp table 3: Numerical results for Olig and TPPP/p25 quantification.
